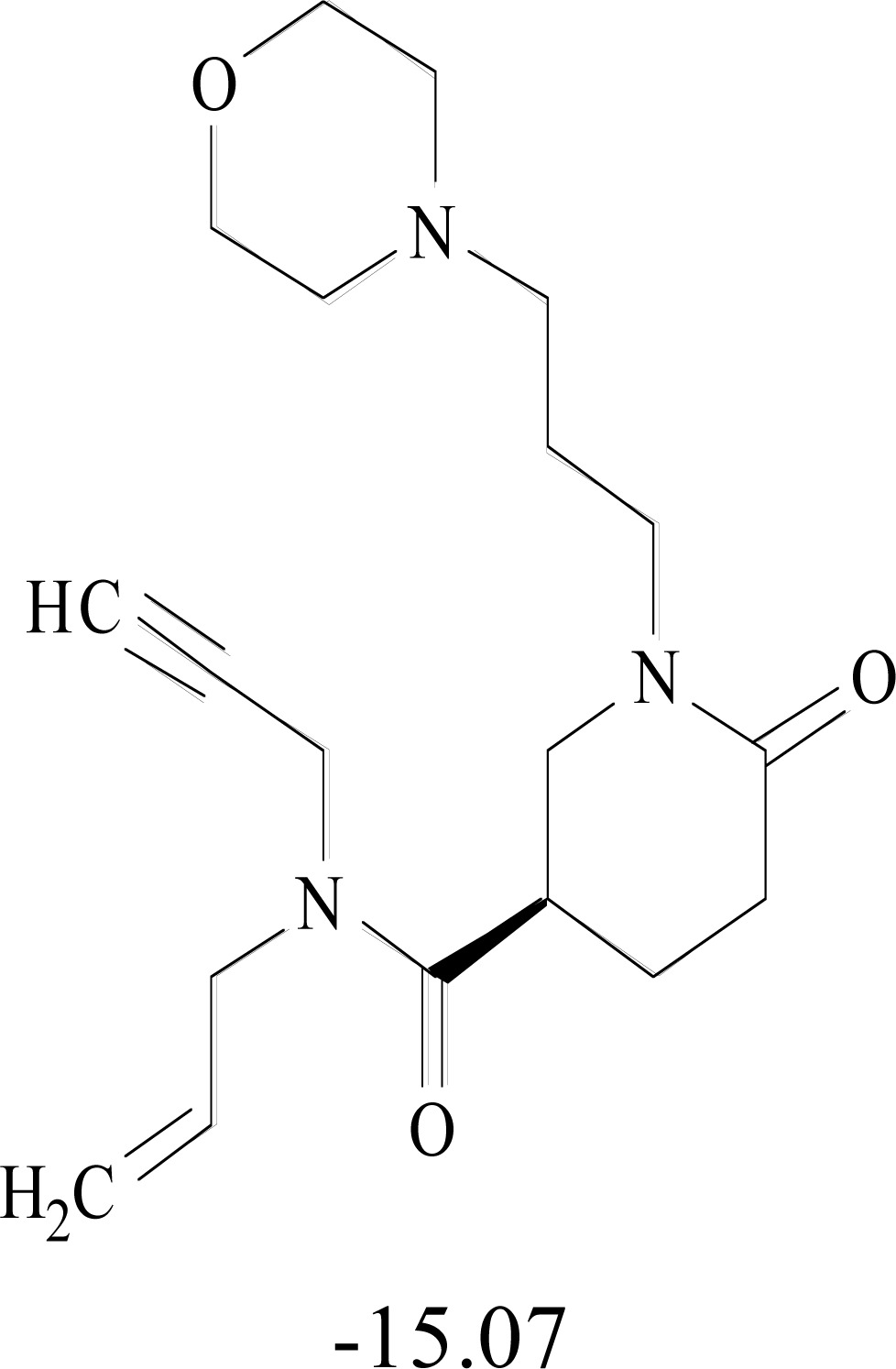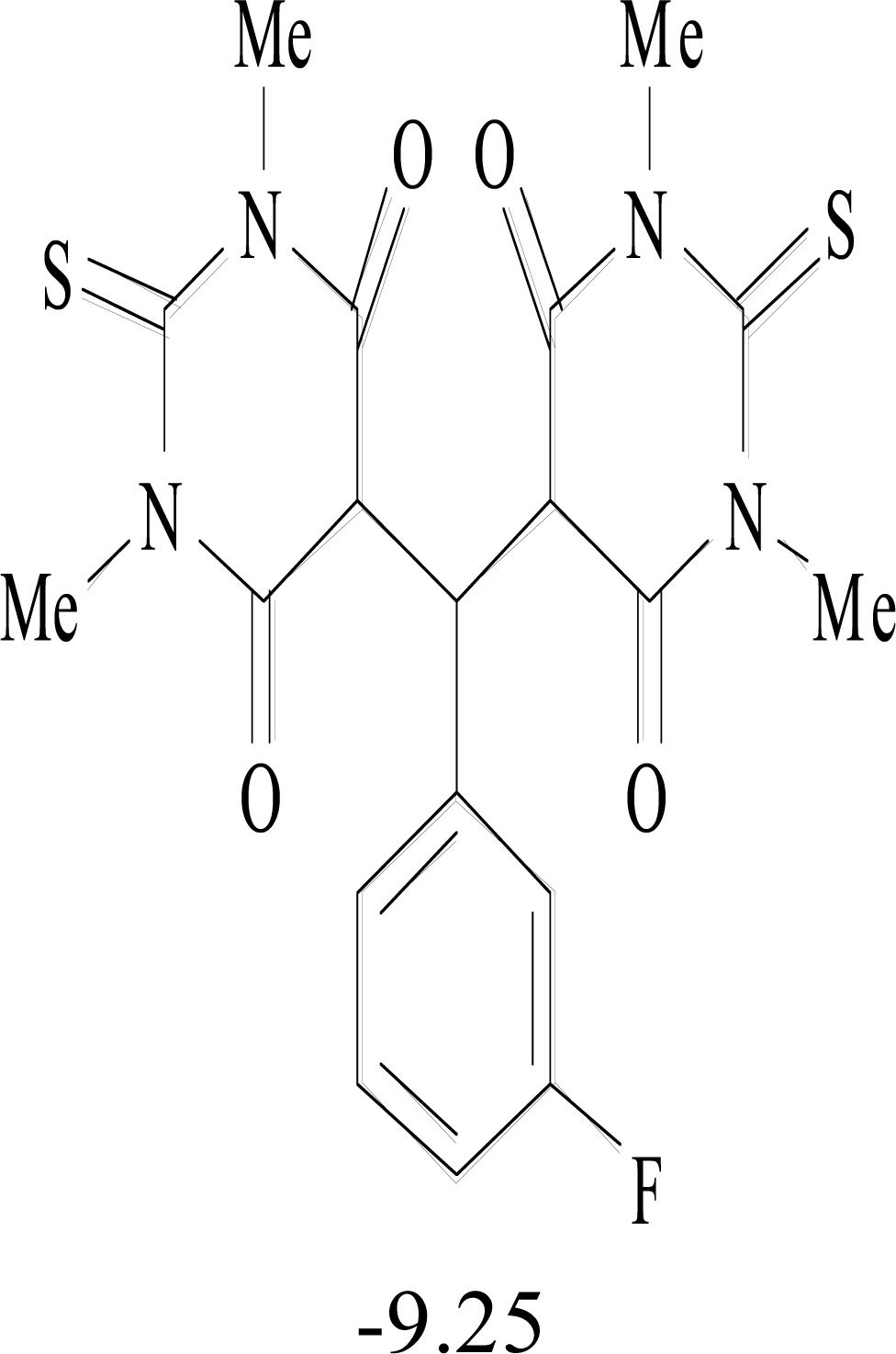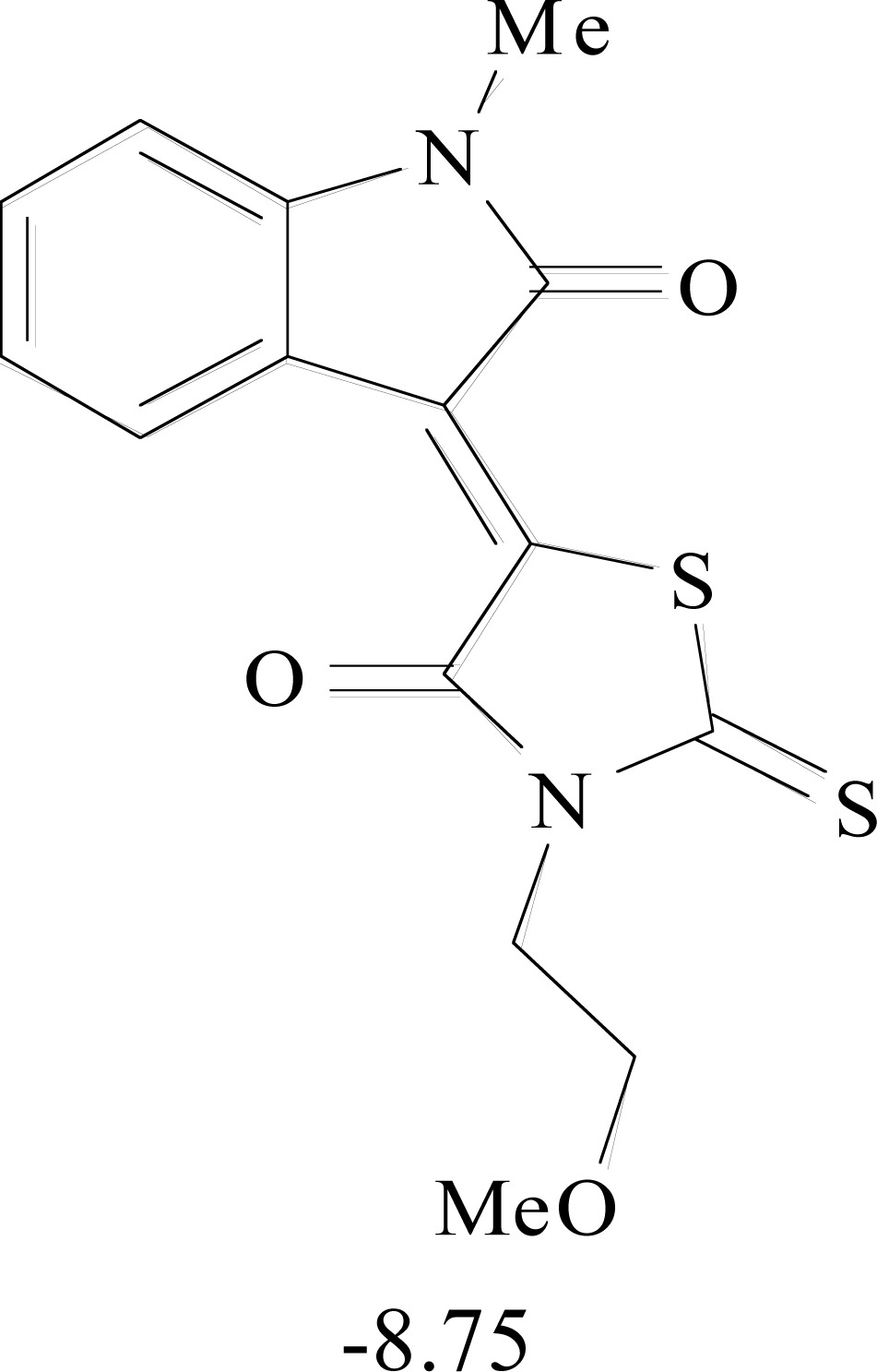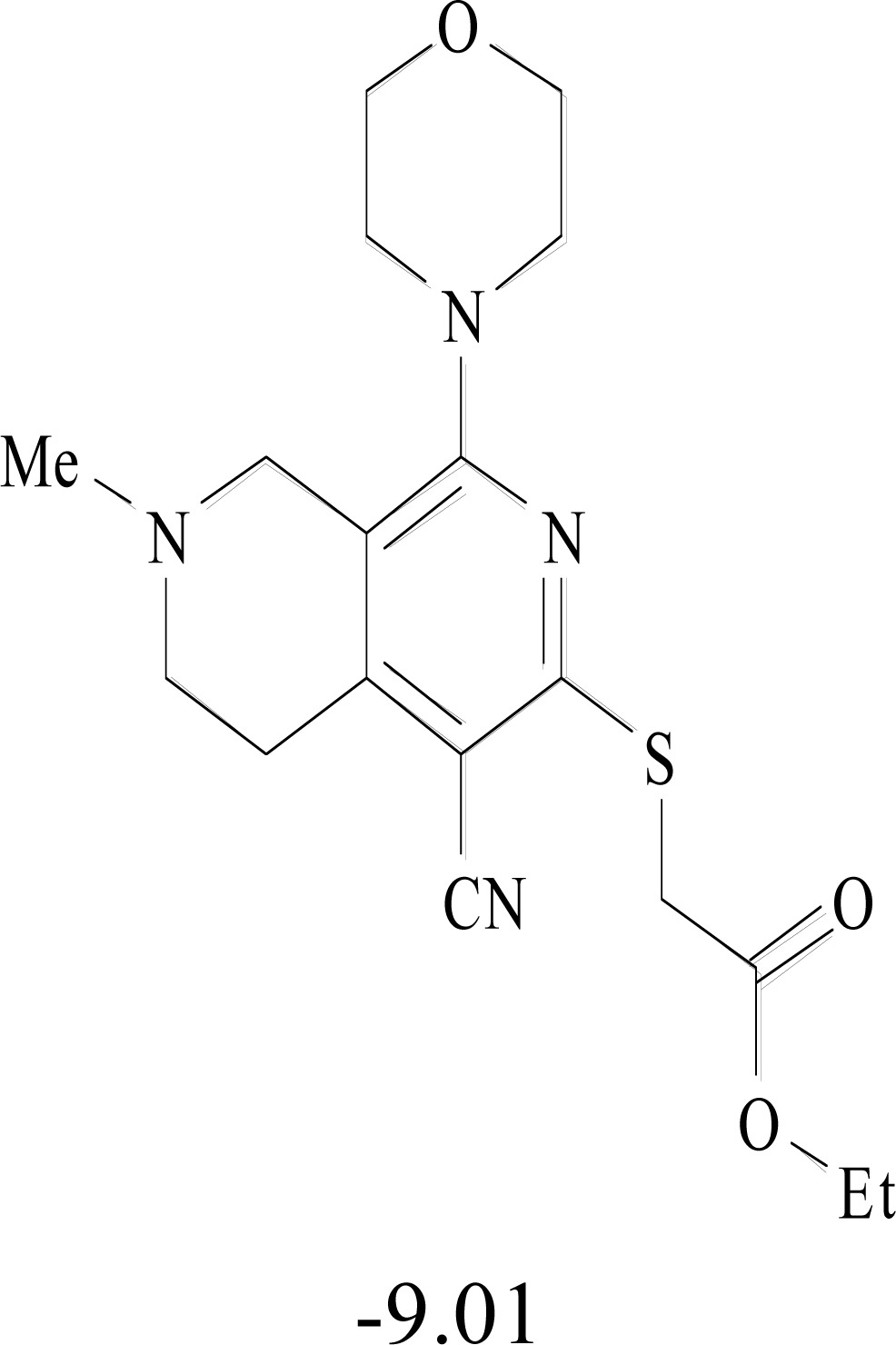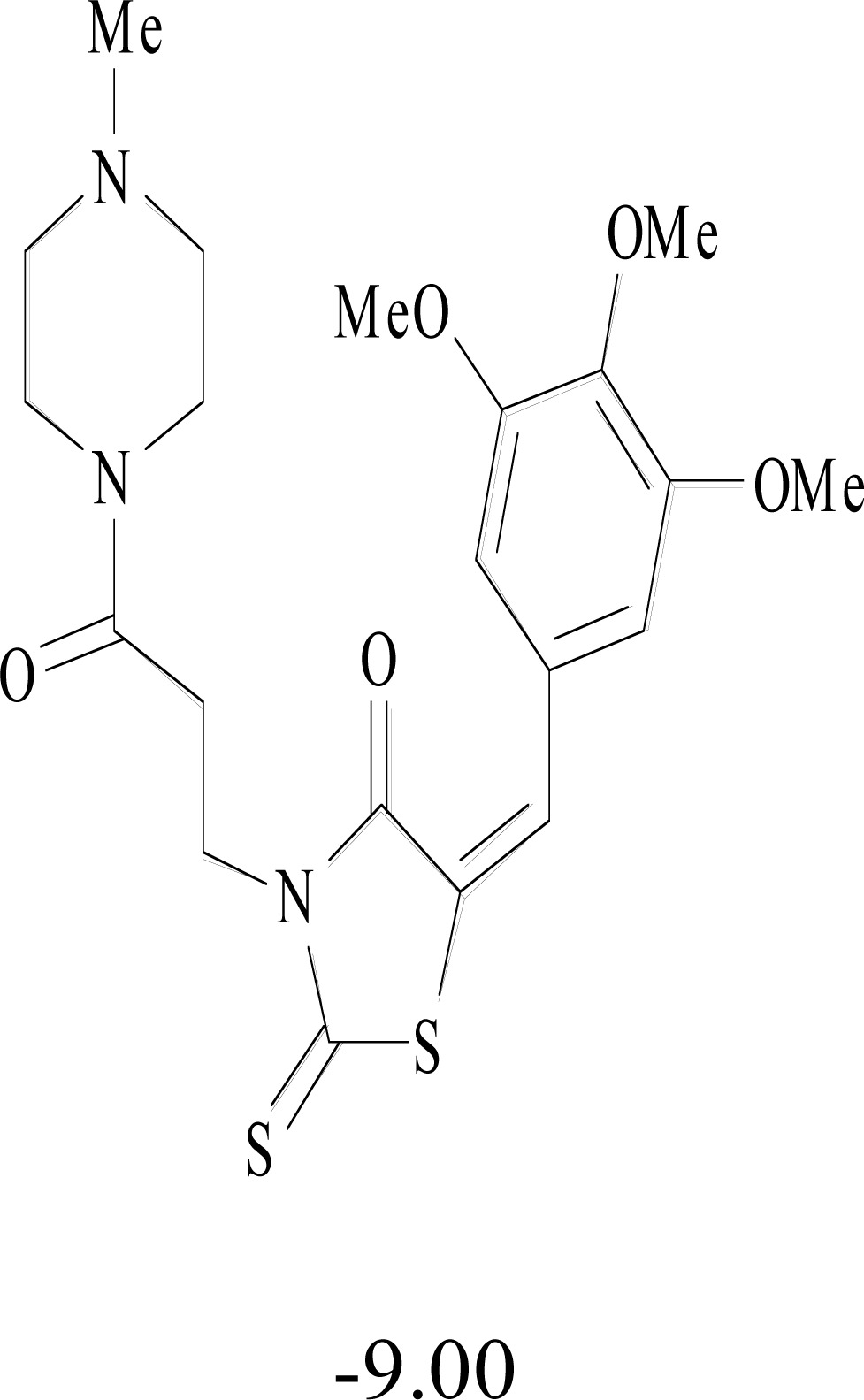# Genomes to Hits *In Silico* - A Country Path Today, A Highway Tomorrow: A Case Study of Chikungunya

**DOI:** 10.2174/13816128113199990379

**Published:** 2013-08

**Authors:** Anjali Soni, Khushhali M Pandey, Pratima Ray, B Jayaram

**Affiliations:** 1 Department of Chemistry & Computational Biology, Indian Institute of Technology, Hauz Khas, New Delhi-110016, India;; 2 Supercomputing Facility for Bioinformatics & Computational Biology, Indian Institute of Technology, Hauz Khas, New Delhi-110016, India;; 3 Department of Chemical Engineering & Biotechnology, Maulana Azad National Institute of Technology, Bhopal-462051, India;; 4 Department of Pediatrics, All India Institute of Medical Sciences, New Delhi-110029, India;; 5 Kusuma School of Biological Sciences, Indian Institute of Technology, Hauz Khas, New Delhi-110016, India

**Keywords:** Genome annotation, protein folding, docking and scoring, lead molecule, CHIKV.

## Abstract

These are exciting times for bioinformaticians, computational biologists and drug designers with the genome and proteome sequences and related structural databases growing at an accelerated pace. The post-genomic era has triggered high expectations for a rapid and successful treatment of diseases. However, in this biological information rich and functional knowledge poor scenario, the challenges are indeed grand, no less than the assembly of the genome of the whole organism. These include functional annotation of genes, identification of druggable targets, prediction of three-dimensional structures of protein targets from their amino acid sequences, arriving at lead compounds for these targets followed by a transition from bench to bedside. We propose here a “Genome to Hits *In Silico*“ strategy (called *Dhanvantari*) and illustrate it on Chikungunya virus (CHIKV). “Genome to hits” is a novel pathway incorporating a series of steps such as gene prediction, protein tertiary structure determination, active site identification, hit molecule generation, docking and scoring of hits to arrive at lead compounds. The current state of the art for each of the steps in the pathway is high-lighted and the feasibility of creating an automated genome to hits assembly line is discussed.

## 1 INTRODUCTION

**Fig. (1):**
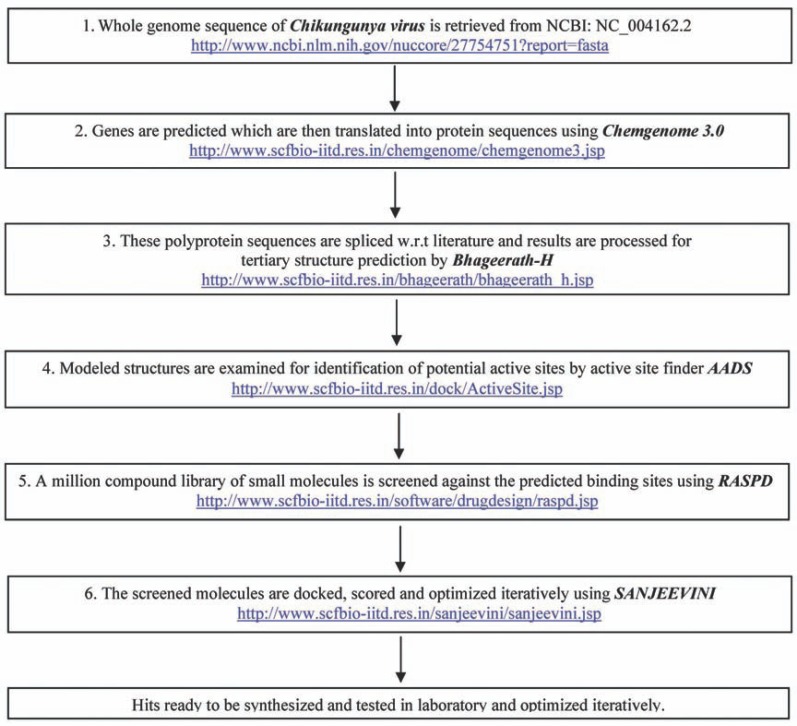
Flow diagram illustrating the steps involved in *Dhanvantari* pathway to achieve hit molecules from genomic information.

The automation of genomes to hit molecules pathway poses several challenges. It involves, *inter alia*, (i) accurate genome annotation, (ii) identification of druggable target proteins, (iii) determination of 3-dimensional structures of protein targets, (iv) identification of hits for the target, (v) optimization of hits to lead molecules to realize high levels of affinity and selectivity to the target and low toxicity. Here, we describe the progresses achieved in each of the above areas, the conceivability of a “Genome to hits” assembly line *in silico *(Fig. **1**) and illustrate the approach with chikungunya virus (CHIKV). 

## 2 BACKGROUND

We describe here the science and the software behind “Genome to Hits” assembly line which comprises six steps (Fig. **1**), classifiable into three major areas of research viz. (a) genome annotation (steps 1 and 2), (b) protein tertiary structure prediction (step 3) and (c) structure based drug design (steps 4 to 6). Information available on chikungunya virus, which is taken up as an illustrative case in this study is summarized in the subsection (d). 

### a Genome Annotation.

The computational genome annotation can play a vital role in finding potential therapeutic target molecules for pathogens. In the present research scenario, it is a big challenge to carry out the structural and functional annotation of the whole genome sequence or the translated ORFs (open reading frames). These annotations can be used in comparative genomics, pathway reconstruction and particularly in drug design. 

Genome annotation is the process of exploring biological/functional information from sequences (Table **1**). It is done by following two main steps: (i) identification of distinct, potentially functional elements on the genome, a process called gene prediction in the context of identification of protein coding regions and (ii) assignment of biological function to these elements (genes or proteins).

Automated annotation tools provide a faster computational annotation as compared to manual annotation (curation) which involves human expertise. Ideally, these approaches coexist and complement each other in the same annotation pipeline. The basic level of annotation involves finding genes and isolating the protein coding sequences from non-coding sequences. A variety of computational approaches have been developed to permit scientists to view and share genome annotations (Table **2**). Most of the available computational methods are knowledge-based and adopt techniques like Hidden Markov Models or machine learning methods. The accuracies of these models are limited by the availability of data on experimentally validated genes, and as typically seen in newly sequenced genomes, can lead to suboptimal levels of prediction. *Ab initio* methods originating in physico-chemical properties of DNA can help overcome the limitations of knowledge-based methods.

Generally for annotation purposes, homologous sequences in protein sequence databases are searched. The state of the art tool for such database searches is PSI-BLAST (Position Specific Iterated Basic Local Alignment Search Tool) [1, 2]. The performance of PSI-BLAST and other database search tools to identify homologs of a given query in a sequence database has been measured by others [3]. However these benchmarks do not suffice the requirements in genome annotation. Our efforts are aimed at eliminating the limitations of PSI-BLAST in correctly annotating protein coding sequences in genomes by using *ab initio* approach. Physico-chemical properties such as hydrogen bonding, stacking, solvation etc. show clear signatures of the functional destiny of DNA sequences [4-8], which has formed the basis of *Chemgenome*. In the present study, we have used *Chemgenome*, the SCFBio tool (http://www.scfbio-iitd.res.in/chemgenome/chemgenome3.jsp) to produce and interpret structural annotations for the viral genome of *Chikungunya virus*. 

### b Protein tertiary structure prediction.

The genome annotation is followed by protein annotation at structural, functional and at genomic scale which is essential for routine work in biology and for any systematic approach to the modeling of biological systems. To bridge the expanding sequence-structure gap, many computational approaches are becoming available which assign structure to a novel protein from its amino acid sequence. A plethora of automated methods to predict protein structure have been developed based on a variety of approaches. These include (a) homology modeling, (b) fold recognition or threading, (c) *ab initio* or *de novo* methods. Homology modeling and fold recognition methods utilize the information derived from structures solved previously via x-ray and NMR methods. This method is effective, popular, reliable and fast for protein tertiary structure prediction when a close sequence homolog exists in the structural repositories. Several protein structure prediction tools are available in the public domain (Table **3**). To make biological sense out of large volumes of sequence data, it is necessary to compare the protein sequences with those proteins that have been already characterized biochemically. To design drug molecules, structural annotation plays an important role. Structural genomics (SG) efforts facilitate such comparisons by determining the structures for a large number of protein sequences, but most SG targets have not been functionally characterized. It is already known that accurate functional details of a protein can neither be inferred from its sequence alone nor from sequence comparisons with other proteins whose structures and functions are known but only from its own native structure [9-11]. 

Several efforts are being made to unravel the physico-chemical basis of protein structures and to establish some fundamental rules of protein folding. Despite the successes, protein tertiary structure prediction still remains a grand challenge - an unsolved problem in computational biochemistry [11, 12-26].

*Ab initio* or *de novo* methods are frequently employed for predicting tertiary structures of proteins by incorporating the basic physical principles, irrespective of the availability of structural homologs. In this study, *Bhageerath* and* Bhageerath-H* servers are employed for protein structure prediction. *Bhageerath* is an energy based software suite for predicting tertiary structures of small globular proteins, available at http://www.scfbio-iitd.res.in/bhageerath/index.jsp [12, 27]. It predicts five candidates for the native, from the input query sequence. *Bhageerath-H* [28] is a hybrid (homology + *ab initio*) server for protein tertiary structure prediction [29, 30]. It identifies regions which show local sequence similarity in respect to sequences in RCSB (protein data bank) to generate 3D fragments which are patched with *ab initio* modeled fragments to generate complete structures of the proteins. This server again predicts the best five energetically favorable structures, which are expected to be close to the native. The knowledge of tertiary structures of proteins serves as a basis for structure-based drug design. 

### c Structure based drug design.

Design of small molecules in structure based drug discovery requires knowledge of the binding pocket on the protein which upon blockade results in loss of function. Experimental information on protein active sites and function loss are useful. In the absence of any experimental information, one could identify all potential binding sites on the protein from the structural information (Table **4**). In this study we use, AADS (http://www.scfbio-iitd.res.in/dock/ActiveSite_new.jsp) methodology for an automated identification of ten potential binding pockets which are expected to bracket the true “active site” (binding pocket). AADS requires the 3D structure of the target protein and detects the top 10 potential binding sites with 100% accuracy in capturing the actual binding (active) site.

Once the binding pockets on proteins are identified, libraries of small molecules are screened against these sites to identify a few hit molecules using software such as RASPD (http://www.scfbio-iitd.res.in/software/drugdesign/raspd.jsp). RASPD protocol is designed in the spirit of structure-based drug design approach but with a rapid turnover rate. RASPD screens small molecule databases against the active sites based on physiochemical descriptors or in general the set of Lipinski parameters such as hydrogen bond donors, hydrogen bond acceptors, molar refractivity, Wiener index and volume for the protein and drug and also the functional groups [31-33]. The most interesting feature of RASPD is that it generates a set of hit molecules based on the complementarities of the afore-mentioned properties with a certain cutoff binding affinity bypassing the conventional docking and scoring strategies, which reduces the search time significantly. The libraries incorporated in RASPD are a million compound library of small molecules and a natural product library. The users can also sketch molecules of their choice or use a non-redundant dataset of small molecules NRDBSM [34] (http://www.scfbio-iitd.res.in/software/nrdbsm/index.jsp) and submit them for RASPD screening. 

The screening is followed by atomic level docking and scoring strategies (Table **5**) such as *Sanjeevini* (http://www.scfbio-iitd.res.in/sanjeevini/sanjeevini.jsp) to identify a few candidates which could be pursued as leads for experimental synthesis and validation [35, 36]. ParDOCK module of *Sanjeevini *is an all-atom energy based Monte Carlo algorithm for protein-ligand docking. It involves the positioning of ligands optimally with best configuration in the protein binding site and scores them based on their interaction energies. This utility is freely accessible at http://www.scfbio-iitd.res.in/dock/pardock.jsp [37]. ParDOCK uses BAPPL scoring function [38] for atomic level scoring of non-metallo protein ligand complexes and in ranking them accurately with their estimated free energies. BAPPL is again freely accessible at http://www.scfbio-iitd.res.in/software/drugdesign/bappl.jsp. The accuracy of this scoring function in predicting binding free energy is high with ±1.02 kcal/mol average error and a correlation coefficient of 0.92 between the predicted and experimental binding energies for 161 protein-ligand complexes. An extended version of BAPPL, i.e. BAPPL-Z can be used for the prediction of binding energies of the complexes having zinc metal ion in their active sites. BAPPL-Z utility is accessible at http://www.scfbio-iitd.res.in/software/drugdesign/bapplz.jsp [39]. All these tools are collectively gathered in *Sanjeevini *software, which is a complete drug design software suite, freely accessible at (http://www.scfbio-iitd.res.in/sanjeevini/sanjeevini.jsp) [34, 40-47]. Thus, the assessment of candidate molecules is done based on their binding energies and the molecules identified as good binders to the target are considered further for synthesis and testing.

### d Chikungunya Virus.

Chikungunya fever (CHIK) is a mosquito (*Aedes aegypti)* borne devastating disease caused by Chikungunya virus (CHIKV), an alphavirus belonging to the family Togaviridae. It is one of the most important re-emerging infectious diseases in Africa and Asia with sporadic intervals and is responsible for significant global impact on public health problems [48-62]. CHIKV is listed as a category C pathogen in 2008 by National Institute of Allergy and Infectious Diseases (NIAID) and as a biosafety level 3 (BSL3) pathogen [50, 63-66]. CHIKV causes debilitating and prolonged arthralgic syndrome incapacitating the affected population for longer periods. CHIKV is usually found in tropics but has widespread across the globe in recent years due to a range of transmission vectors, globalization and climatic changes [67-111]. The ‘Chikungunya’ word has originated from the Makonde root verb kungunyala, meaning “that which bends up” [112, 113] which is in reference to drying up or becoming contorted and signifies the cause of stooped posture developed due to the excruciating joint and muscle pain and other rheumatologic manifestations [114, 115]. The disease etiology consists of sudden onset of fever with arthalgia, which generally resolves within a few days [116, 117].

**Fig. (2):**
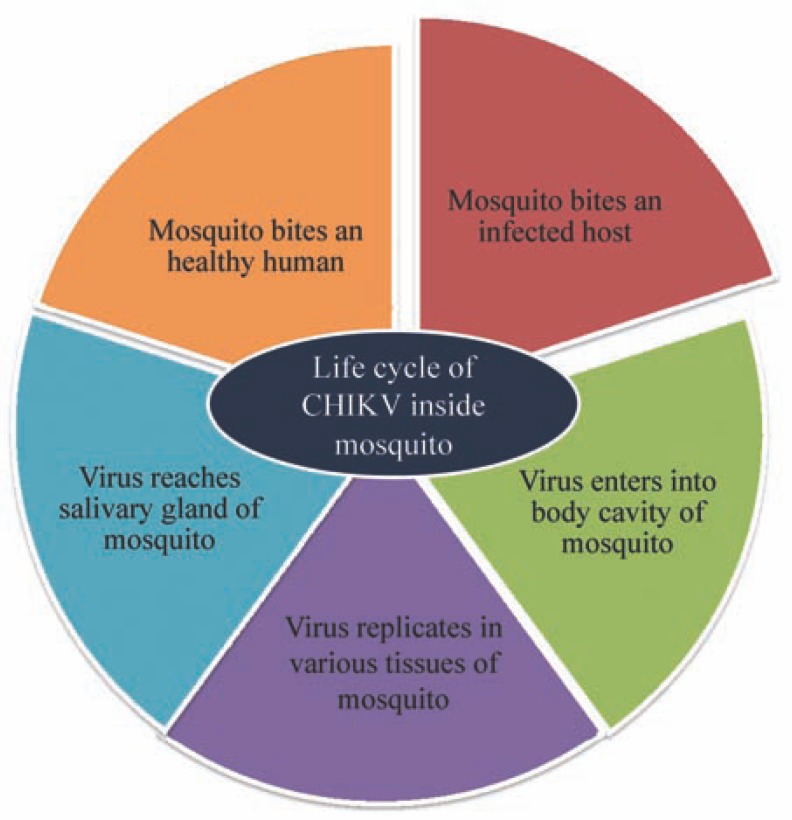
Flow diagram depicting the movement of CHIKV from veremic host to mosquito and from mosquito to healthy host.

Female mosquitoes acquire the virus by taking blood from viremic vertebrate hosts (Fig. **2**). The virus elicits a persistent infection and replicates at a high pace, especially in the salivary glands of the insects [118, 119]. In addition to salivary glands, it replicates in various other organs inside body cavity including gut, ovary, neural tissue, body fat etc. [120]. When this CHIKV loaded mosquito infects a healthy human, it transfers the virus into its blood stream. These virions through interaction with the receptors reach the target cells by endocytosis. The acidic environment of the endosome triggers conformational changes in the viral envelope that expose the E1 peptide [121-125], which mediates virus-host cell membrane fusion. This allows cytoplasmic delivery of the core and release of the viral genome in cytoplasm. The site of mRNA transcription is in the cell cytoplasm.

CHIKV is an enveloped, spherical bodied virus of about 70nm in diameter. The virion genome consists of a linear single-stranded (ss), positive-sense RNA molecule of approximately 11.8 kb length, where the 5’ end is capped with a 7-methylguanosine while the 3’ end is polyadenylated. The CHIKV genome is comprised of 30% A, 25% C, 25% G and 20% T (U) base pairs with two long open reading frames (ORF) that encode the non-structural (2474 amino acids) and structural polyproteins (1244 amino acids) [126-131]. 

**Fig. (3):**
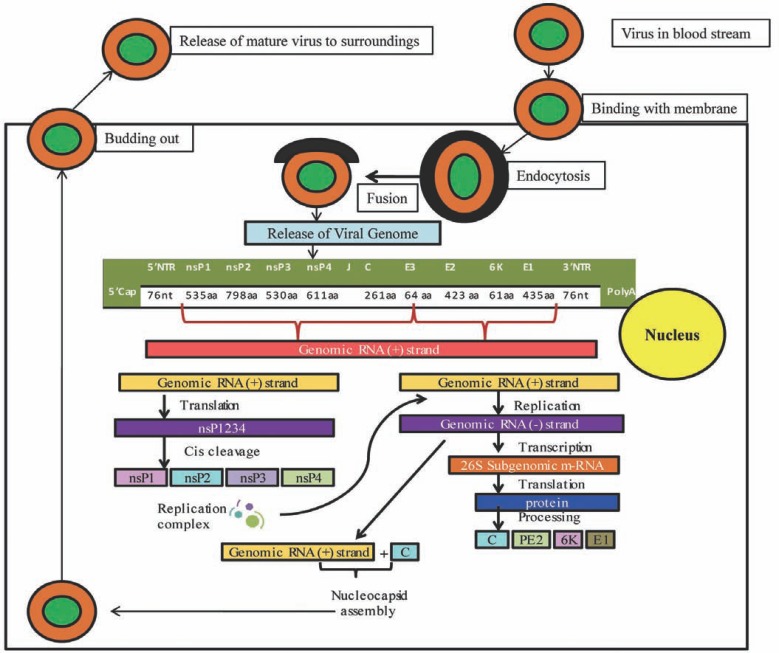
The lifecycle of CHIKV in the host cell.

The genomic organization of CHIKV is considered to be 5’cap-nsP1-nsP2-nsP3-nsP4-(junction)-C-E3-E2-6K-E1-Poly (A)3’ (Fig. **3**). The non-structural polyproteins (nsP1-4) located in an ORF of 7425 nucleotides get initiated by a start codon at position 77-79 and terminated by a stop codon at position 7499-7501. This polyprotein is autocatalytically cleaved to produce nonstructural proteins nsP1, nsP2, nsP3 and nsP4. In contrast, the structural polyproteins are located on an ORF of 3735 nucleotides with a start codon at position 7567-7569 and a stop codon at position 11299-11313. Likewise, this polyprotein is cleaved to produce the structural proteins namely the capsid protein (C), the glycoproteins E1, E2 and E3 and 6K [126, 132-136]. The polypeptides are cleaved into active proteins by viral and cellular proteases [137-148]. The functional properties of the active cleaved proteins are summarized in (Table **6**).

Although no specific drugs are available, CHIK is usually treated with non-steroidal anti-inflammatory drugs (NSAIDs), with inconsistent success [149-172] (Table **7**). Owing to the non-availability of a potential drug to cure the disease, there is an urgent need to adopt a skilled strategy to develop new therapeutics. We describe in the following section how computational approaches can help in reducing the time in arriving at potential lead molecules. 

## 3 CALCULATIONS & RESULTS: APPLICATION OF THE G2H ASSEMBLY LINE TO CHIKV

The genome sequence of Chikungunya virus was retrieved from NCBI (http://www.ncbi.nlm.nih.gov/nuccore/NC_004162). For gene prediction, the sequence was processed using *ChemGenome 3.0* (http://www.scfbio-iitd.res.in/chemgenome/chemgenome3.jsp) software [5, 6]. The results displayed the existence of two genes which were similar to the already published ones, essentially implying that in this case, 100% accuracy is achieved with *ChemGenome 3.0*. These nucleotide sequences were translated to protein sequences by *ChemGenome 3.0*. The proteins in CHIKV are polyproteins i.e. the sequence displayed in results contains sequences for all proteins coded by the gene. The individual proteins from polyprotein are cleaved during post translational processing. Till date no reliable computational approach is available to cleave the polyproteins, therefore the sequences were dissected manually for each protein, based on literature and experimental evidence to identify cleavage site. The *ChemGenome 3.0 *results are archived at http://www.scfbio-iitd.res.in/software/chemgenomeresult.jsp.

**Fig. (4):**
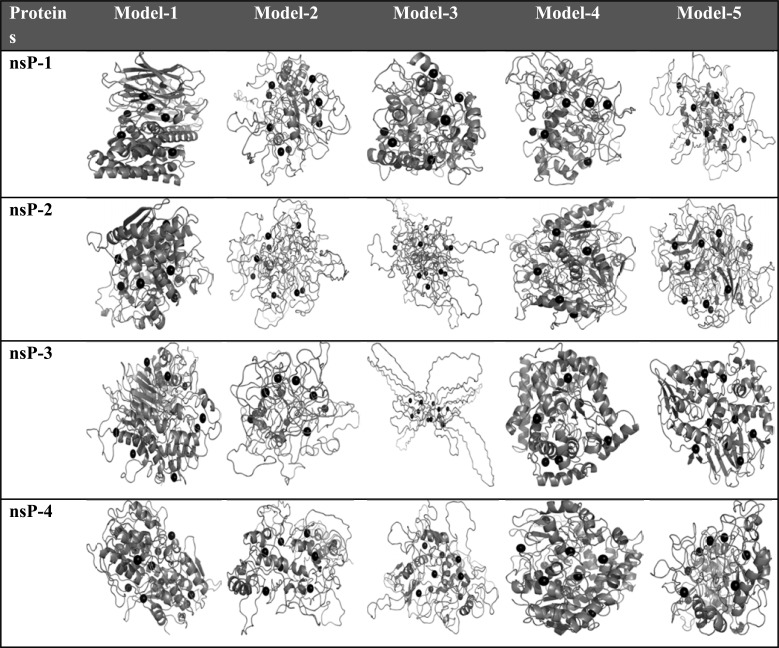
An illustration of the protein structures of CHIKV predicted by *Bhageerath*-H shown along with their binding pockets.

The sequences extracted from *Chemgenome 3.0* served as inputs to *Bhageerath-H* (http://www.scfbio-iitd.res.in/bhageerath/bhageerath_h.jsp), a tertiary structure prediction server [28]. For each submitted sequence, five structures were returned by the server. The results received from *Bhageerath-H* are shown in (Fig. **4**). As no homolog information is available to give strength to these structural models, all the five structures are considered as plausible candidates for the native, and considered for further studies. It may be noted that tertiary structure prediction of structural proteins associated with membranes is a nascent area with low success rate at this stage and hence the focus here has been on nonstructural proteins which can fold autonomously.

Most of the experimentally determined structures have some information of ligand binding domain/site but in the present scenario, CHIKV proteins lack the structural information, thus necessitating detection of ligand binding sites (active sites). In order to facilitate active site detection, an automated version of active site finder i.e. AADS (Automated active site docking and scoring) (http://www.scfbio-iitd.res.in/dock/ActiveSite_new.jsp) is utilized which predicts the potential binding site(s) and further performs the docking of the selected molecule to the top ten cavities in an automated mode [40]. Binding sites on each of the five structural models of each nonstructural protein are identified. Not all cavities determined by the active site identifier may be true binding sites with functional implication but one among them is very likely to be such a site. The additional cavities can be checked for their ability to act as allosteric sites. The predicted top 10 binding sites are shown as black dots in the protein structures (Fig. **4**). 

In search of probable hits, the 10 cavities per structure identified by AADS are further subjected to RASPD (Rapid screening of preliminary drugs) (http://www.scfbio-iitd.res.in/software/drugdesign/raspd.jsp) software [41]. The RASPD returned more than 500 molecules against the predicted cavities of CHIKV proteins with -8.00 kcal/mol as the binding energy cutoff. 

The *in silico* drug design beyond this stage involves rigorous docking and scoring [173, 174]. The hits identified from screening via RASPD above are further docked with their respective target site using *Sanjeevini* software (http://www.scfbio-iitd.res.in/sanjeevini/sanjeevini.jsp) which utilizes ParDOCK as a docking tool. For all the modeled structures, one molecule for each cavity has been proposed on SCFBio’s CHIKV webpage which is accessible at http://www.scfbio-iitd.res.in/software/chikv.jsp. This webpage contains information on the genome annotation, protein tertiary structure prediction, and hit molecule identification and docking and scoring results of the complete genome to hit protocol. 

The best 20 molecules selected against the nonstructural proteins of CHIKV are displayed in (Table **8**). From here on, the *in silico* strategies go hand-in-hand with experimentation. In an iterative process of synthesis, testing, modification, docking and scoring, these molecules can be further improved to yield candidate drugs while taking care of the ADMET profiles [175-180].

## 4 DISCUSSION ON THE G2H ASSEMBLY LINE

The wealth of information available from experimental host-pathogen interaction studies invites computational biologists to develop databases and newer computational methods to advance further focused experimentation. Consequently, bioinformatics is rapidly evolving into independent fields addressing specific problems in interpreting (i) genomic sequences, (ii) protein sequences and 3D-structures, as well as (iii) transcriptome and macromolecular interaction data. It is thus increasingly difficult for the biologist to choose the computational approaches that perform best in inhibiting the growth of pathogen in the host. 

A basic overview of the G2H technology is given in this review with an application to Chikungunya virus. G2H assembly line is a culmination of several recent advances in computational chemistry and computational biology implemented in a high performance computing environment. At least three areas for further improvement can be immediately identified: (i) development of algorithms for cleavage of polyproteins, (ii) algorithms for identification of druggable protein targets, (iii) improved accuracies in tertiary structure prediction of nonstructural proteins, (iv) development of methods for determining tertiary structures of structural proteins and (v) identification of hit molecules with reduced toxicities. This protocol should ultimately result in an accelerated emergence of new methods for treating infectious diseases. Similarly, metabolic disorders can also be accessed via the “Genome to Hit” pathway. 

## 5 CONCLUSION & PERSPECTIVES

Post-genomic research era encompasses many diverse aspects of modern science. The “Genome to hits” pathway described here symbolizes the emergence of an integrated technology to address specific health issues, and more specifically provides a novel and rapid approach to identifying new and potent hit molecules from genomic information. 

## SUPPLEMENTARY INFORMATION ON CHIKUNGUNYA AT SCFBIO WEBSITE

Details of the results on genes and protein tertiary structures predicted, binding pockets, hit molecules identified and lead molecules proposed for synthesis are available for free download from the SCFBio website (http://www.scfbio-iitd.res.in/software/chikv.jsp). These results will be updated periodically with improvements in protocols for protein structure prediction and ADMET evaluations.

## Figures and Tables

**Table 1.** Some Typical Features Considered During Genome Annotation

Genome Annotation	
***Structural annotation: ***identification of genomic elements	***Functional annotation: ***assigning biological information to genomic elements
ORFs and their localization gene structure coding regions regulatory motifs	biochemical function biological function involved in regulation and interactions control of expression

**Table 2.** List of Tools Available for Gene Prediction

Sl. No.	Softwares	URLs	Methodology
1.	FGENESH	http://linux1.softberry.com/all.htm	*Ab initio*
2.	GeneID	http://www1.imim.es/geneid.html	*Ab initio*
3.	GeneMark	http://exon.gatech.edu/GeneMark/gmchoice.html	*Ab initio*
4.	GeneMark.hmm	http://exon.gatech.edu/hmmchoice.html	*Ab initio*
5.	GeneWise	http://www.ebi.ac.uk/Tools/Wise2/	Homology
6.	GENSCAN	http://genes.mit.edu/GENSCAN.html	*Ab initio*
7.	Glimmer	http://www.tigr.org/software/glimmer/	*Ab initio*
8.	GlimmerHMM	http://www.cbcb.umd.edu/software/glimmerhmm/	*Ab initio*
9.	GRAILEXP	http://compbio.ornl.gov/grailexp	*Ab initio*
10.	GENVIEW	http://zeus2.itb.cnr.it/~webgene/wwwgene.html	*Ab initio*
11.	GenSeqer	http://bioinformatics.iastate.edu/cgi-bin/gs.cgi	Homology
12.	PRODIGAL	http://prodigal.ornl.gov/	Homology
13.	MORGAN	http://www.cbcb.umd.edu/~salzberg/morgan.html	*Ab initio*
14.	PredictGenes	http://mendel.ethz.ch:8080/Server/subsection3_1_8.html	Homology
15.	MZEF	http://rulai.cshl.edu/software/index1.htm	*Ab initio*
16.	Rosetta	http://crossspecies.lcs.mit.edu	Homology
17.	EuGéne	http://eugene.toulouse.inra.fr/	*Ab initio*
18.	PROCRUSTES	http://www.riethoven.org/BioInformer/newsletter/archives/2/procrustes.html	Homology
19.	Xpound	http://mobyle.pasteur.fr/cgi-bin/portal.py?#forms::xpound	*Ab initio*
20.	Chemgenome	http://www.scfbio-iitd.res.in/chemgenome/chemgenome3.jsp	*Ab initio*
21.	Augustus	http://augustus.gobics.de/	*Ab initio*
22.	Genome Threader	http://www.genomethreader.org/	Homology
23.	HMMgene	http://www.cbs.dtu.dk/services/HMMgene/	*Ab initio*
24.	GeneFinder	http://people.virginia.edu/~wc9c/genefinder/	*Ab initio*
25.	EGPRED	http://www.imtech.res.in/raghava/egpred/	*Ab initio*
26.	mGene	http://mgene.org/web	*Ab initio*

**Table 3.** List of Tools Available for Protein Tertiary Structure Prediction

Sl. No	Softwares	URLs	Description
1.	CPHModels3.0	http://www.cbs.dtu.dk/services/CPHmodels/	Protein homology modeling server
2.	SWISS-MODEL	http://swissmodel.expasy.org/SWISS-MODEL.html	A fully automated protein structure homology-modeling server
3.	Modeller	http://salilab.org/modeller/	Program for protein structure modeling by satisfaction of spatial restraints
5.	3D-JIGSAW	http://3djigsaw.com/	Server to build three-dimensional models for proteins based on homologues of known structure
6.	PSIPRED	http://bioinf.cs.ucl.ac.uk/psipred/	A combination of methods such as sequence alignment with structure based scoring functions and neural network based jury system to calculate final score for the alignment
7.	3D-PSSM	http://www.sbg.bio.ic.ac.uk/~3dpssm/index2.html	Threading approach using 1D and 3D profiles coupled with secondary structure and solvation potential
8.	ROBETTA	http://robetta.bakerlab.org	*De novo *Automated structure prediction analysis tool used to infer protein structural information from protein sequence data
9.	PROTINFO	http://protinfo.compbio.washington.edu/	*De novo *protein structure prediction web server utilizing simulated annealing for generation and different scoring functions for selection of final five conformers
10.	SCRATCH	http://scratch.proteomics.ics.uci.edu/	Protein structure and structural features prediction server which utilizes recursive neural networks, evolutionary information, fragment libraries and energy
11.	I-TASSER	http://zhanglab.ccmb.med.umich.edu/I-TASSER/	Predicts protein 3D structures based on threading approach
12.	BHAGEERATH	http://www.scfbio-iitd.res.in/bhageerath/index.jsp	Energy based methodology for narrowing down the search space of small globular proteins
13.	BHAGEERATH-H	http://www.scfbio-iitd.res.in/bhageerath/bhageerath_h.jsp	A Homology *ab-initio* Hybrid Web server for Protein Tertiary Structure Prediction

**Table 4.** List of Software Available for Active Site Prediction

S.No	Software	URL	Description
1	SitesIdentify	http://www.manchester.ac.uk/bioinformatics/sitesidentify/	Sequence and geometry based
2	PAR-3D	http://sunserver.cdfd.org.in:8080/protease/PAR_3D/index.html	Structure based
3	FUZZY-OIL-DROP	http://www.bioinformatics.cm-uj.krakow.pl/activesite/	Fuzzy oil drop model
4	CASTp	http://cast.engr.uic.edu	Structure based
5	Pocket-Finder	http://www.modelling.leeds.ac.uk/pocketfinder/	Energy based
6	Q-site finder	http://www.modelling.leeds.ac.uk/qsitefinder/	Energy based
7	PASS	http://www.ccl.net/cca/software/UNIX/pass/overview.shtml	Structure based
8	SURFNET	http://www.biochem.ucl.ac.uk/~roman/surfnet/surfnet.html	Structure based
9	LIGSITE*^CSC^*	http://projects.biotec.tu-dresden.de/pocket/	Based on Connolly surface
10	VOIDOO	http://xray.bmc.uu.se/usf/voidoo.html	Structure based
11	LiGandFit	http://www.phenix-online.org/documentation/ligandfit.htm	Structure based
12	Active site prediction	http://www.scfbio-iitd.res.in/dock/ActiveSite.jsp	Structure based
13	AADS	http://www.scfbio-iitd.res.in/dock/ActiveSite_new.jsp	Structure based
14	Fpocket	http://fpocket.sourceforge.net/	Based on Voronoi tessellation
15	Pocket Picker	http://gecco.org.chemie.uni-frankfurt.de/pocketpicker/index.html	
16	IsoCleft	http://bcb.med.usherbrooke.ca/isocleftfinder.php	graph-matching-based method
17	metaPocket	http://sysbio.zju.edu.cn/metapocket/	Structure based
18	LIGSITEc*^CS^*	http://gopubmed2.biotec.tu-dresden.de/cgi-bin/index.php	Structure based
19	GHECOM	http://strcomp.protein.osaka-u.ac.jp/ghecom/	Structure based
20	ConCavity	http://compbio.cs.princeton.edu/concavity/	Structure based
21	POCASA	http://altair.sci.hokudai.ac.jp/g6/Research/POCASA_e.html	Structure based

**Table 5.** A list of Softwares for Drug Design

Sl. No.	Softwares	URL	Description
1	Discovery studio	http://accelrys.com/products/discovery-studio/structure-based-design.html	Molecular modeling and *de novo* drug design
2	Sybyl	http://www.tripos.com/	Computational software for drug discovery
3	Bio-Suite	http://www.staff.ncl.ac.uk/p.dean/Biosuite/body_biosuite.html	Tool for Drug Design, structural analysis and simulations
4	Molecular Operating Environment (MOE)	http://www.chemcomp.com/	Structure-based drug design, molecular modeling and simulations
5	Glide	https://www.schrodinger.com/products/14/5	Ligand-receptor docking
6	Autodock	http://autodock.scripps.edu/	Protein-ligand docking
7	DOCK	http://dock.compbio.ucsf.edu/	Protein-ligand docking
8	*Sanjeevini*	http://www.scfbio-iitd.res.in/sanjeevini/sanjeevini.jsp	A complete software suite for structure-based drug design
9	ArgusLab	http://www.arguslab.com/arguslab.com/ArgusLab.html	Ligand-receptor docking
10	eHITS	http://www.simbiosys.ca/ehits/index.html	Ligand-receptor docking
11	FlexX	http://www.biosolveit.de/FlexX/	Ligand-receptor docking
12	FLIPDock	http://flipdock.scripps.edu/	Ligand-receptor docking
13	FRED	http://www.eyesopen.com/oedocking	Ligand-receptor docking
14	GOLD	http://www.ccdc.cam.ac.uk/products/life_sciences/gold/	Protein-ligand docking
15	ICM-Docking	http://www.molsoft.com/docking.html	Protein-ligand docking
16	PLANTS	http://www.tcd.uni-konstanz.de/research/plants.php	Protein-ligand docking
17	Surflex	http://www.biopharmics.com/	Protein-ligand docking

**Table 6.** Functional Properties of Structural and Non-structural Proteins Found in *Chikungunya*

Protein Type	Proteins	Functions
NonStructural Proteins NP_690588.1	nsP1	Viral methyl transferase domain (acts as cytoplasmic capping enzyme and transfers 7-methyl-GMP complex to mRNA, thus forming the cap structure)
	nsP2	Viral RNA helicase domain and RNA trisphosphatase (part of the RNA polymerase complex) Peptidase C9 domain (cleaves four mature proteins from non structural polyprotein)
	nsP3	Processing domain (crucial for minus strand and subgenomic 26S mRNA synthesis)
	nsP4	Viral RNA dependent RNA polymerase domain (replicates genomic and antigenomic RNA and also transcribes 26S subgenomic mRNA which encodes for structural proteins)
Structural proteins NP_690589.2	C	Peptidase_S3 domain (autocatalytic cleavage) Trypsin like serine protease domain
	E3	Alphavirus E3 spike glycoprotein domain (tentative)
	E2	Alphavirus E2 glycoprotein domain (virus attachment to host) Transmembrane domain
	6K	Alphavirus E1 glycoprotein domain (virus glycoprotein processing and membrane permeabilization) Signal peptide domain Transmembrane domain
	E1	Alphavirus E1 glycoprotein domain (class II viral fusion protein) Glycoprotein E dimerization domain (forms E1-E2 heterodimer in inactive state and E1 trimerizes in active state) Immunoglobulin E set domain Transmembrane domain

**Table 7.** A list of Drugs Available for Treating Chikungunya Fever

Drug	Category	Description
Chloroquine	Antirheumatic Agents / Antimalarials / Amebicides	It is believed to inhibit the heme polymerase activity
Aspirin	Anticoagulants / cyclooxygenase(COX) Inhibitors / PlateletAggregation Inhibitors	Irreversibly inhibits the activity of both types of cyclooxygenase (COX-1 and COX-2)
Ibuprofen	Anti-inflammatory Agents / COX Inhibitors / Analgesics / Nonsteroidal Anti-inflammatory Agents (NSAIAs)	A non-selective inhibitor of cyclooxygenase, an enzyme invovled in prostaglandin synthesis via the arachidonic acid pathway
Naproxen	COX Inhibitors / Gout Suppressants	It is believed to be associated with the inhibition of cyclooxygenase activity
Ribavirin	Antiviral Agents / Antimetabolites	A potent competitive inhibitor of inosine monophosphate (IMP) dehydrogenase, viral RNA polymerase and messenger RNA (mRNA) guanylyl trasferase (viral); may get incorporated into RNA in RNA viral species.
Prednisolone	Hormonal Glucocorticoids	The antiinflammatory actions of glucocorticoids are thought to involve phospholipase A2 inhibitory proteins, lipocortins
Acetaminophen	Analgesics, Non-Narcotic / Antipyretics	Inhibits various forms of cyclooxygenase, COX-1, COX-2, and COX-3 enzymes

**Table 8.** Structural Representations of 20 Molecules Showing high Affinity to the Nonstructural Proteins of CHIKV. (Computed Binding Energies are also Shown in kcal/mol Underneath Each Molecule)

Protein	Model-1	Model-2	Model-3	Model-4	Model-5
nsP1	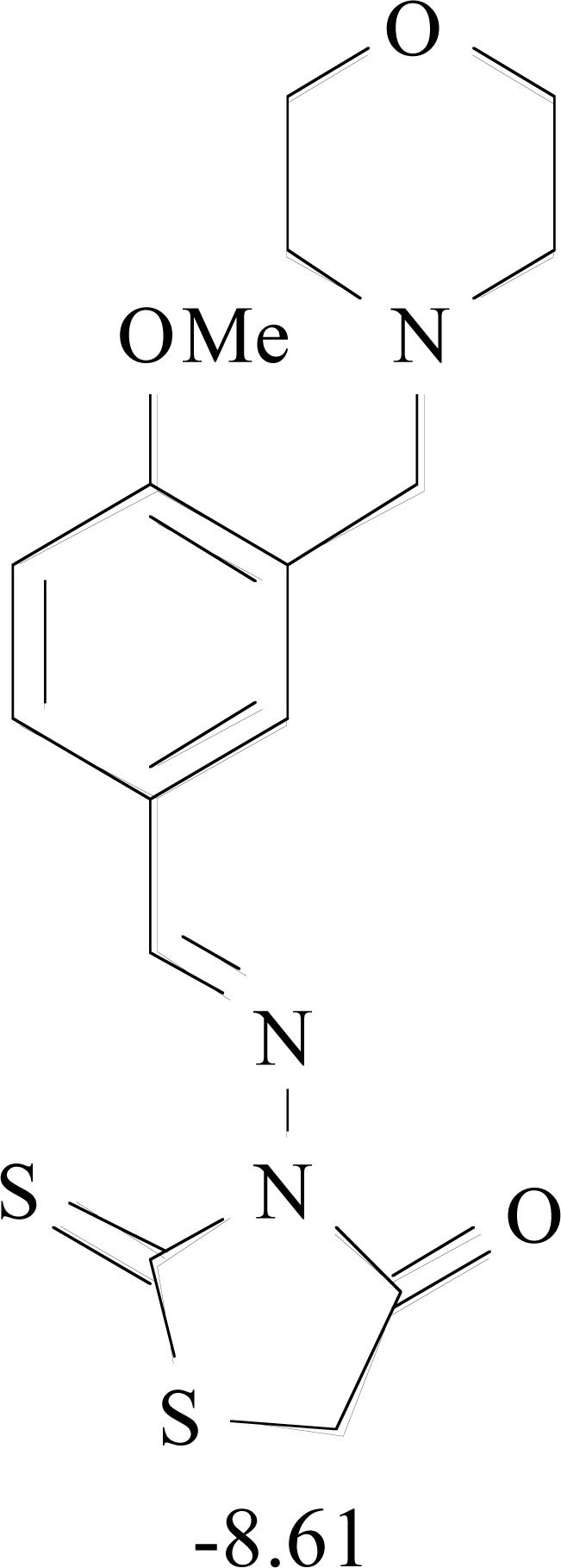	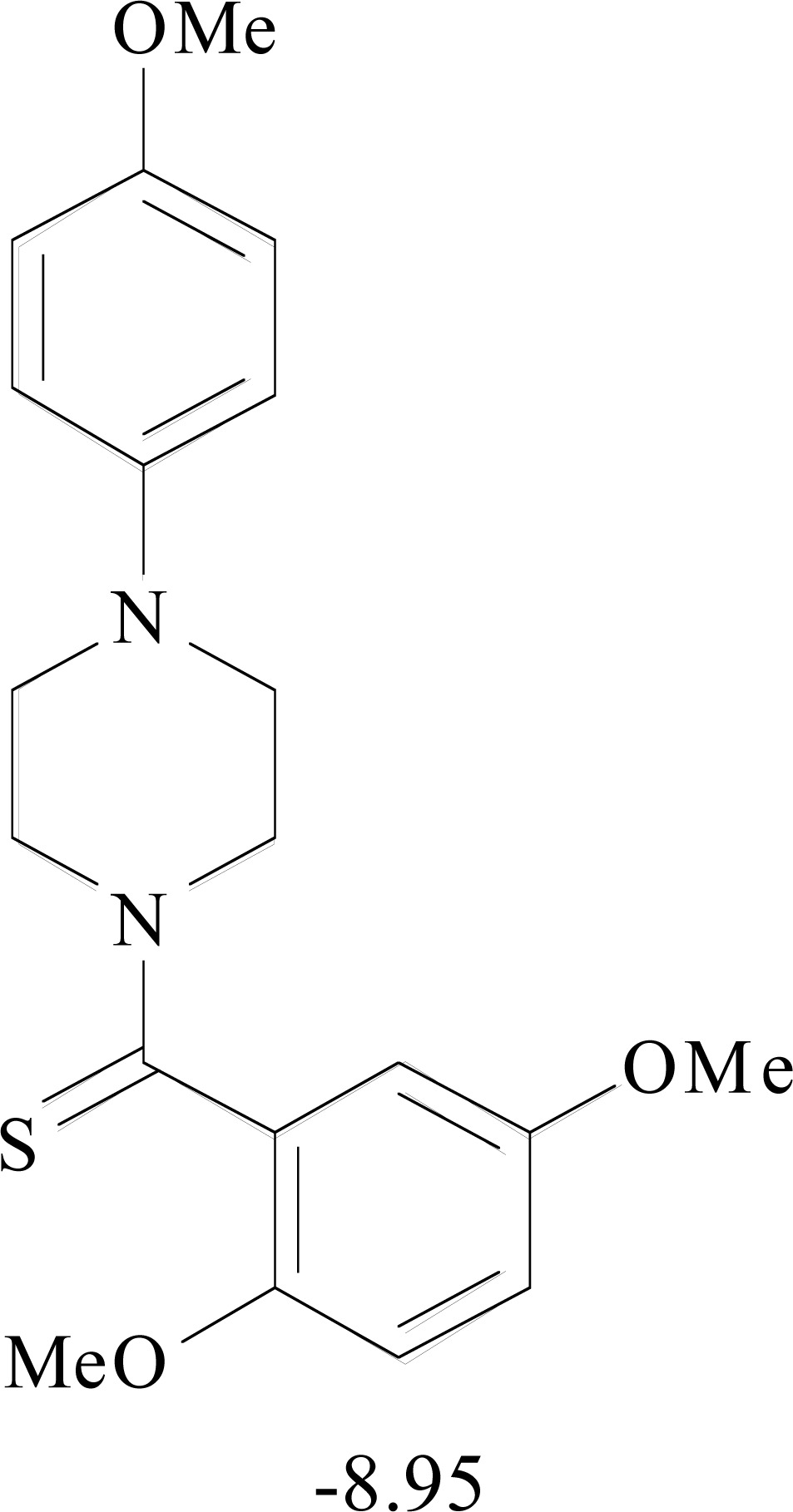	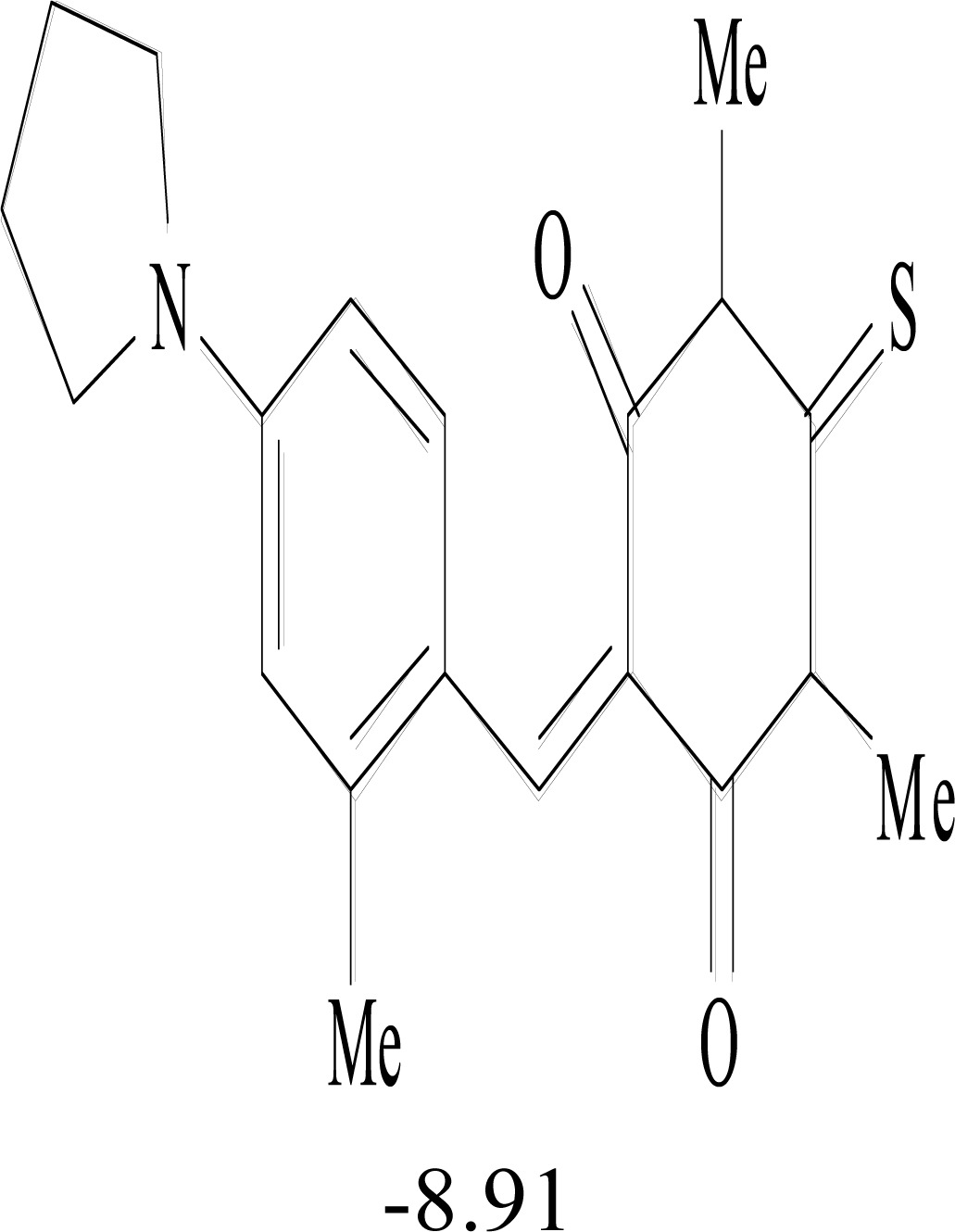	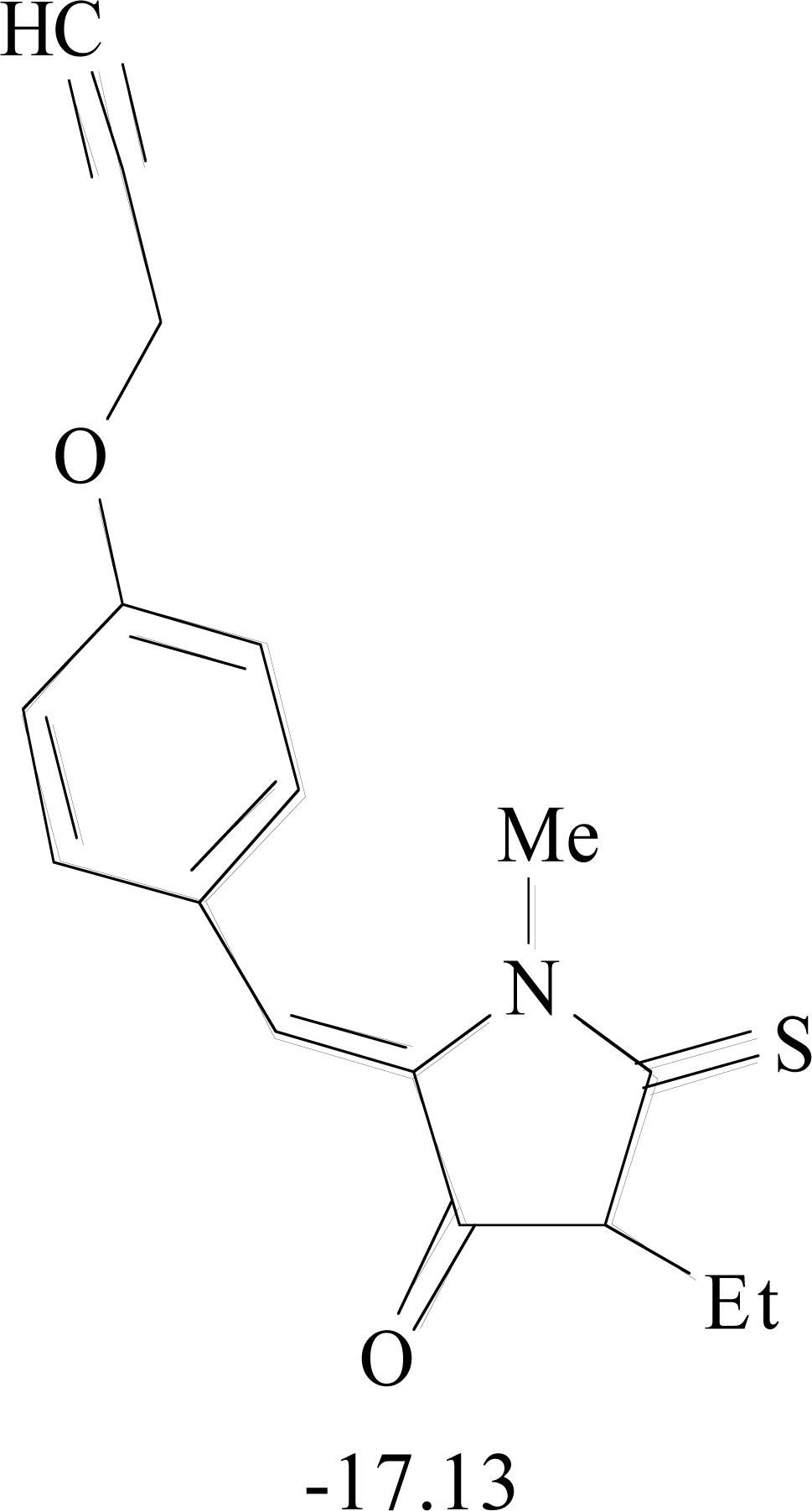	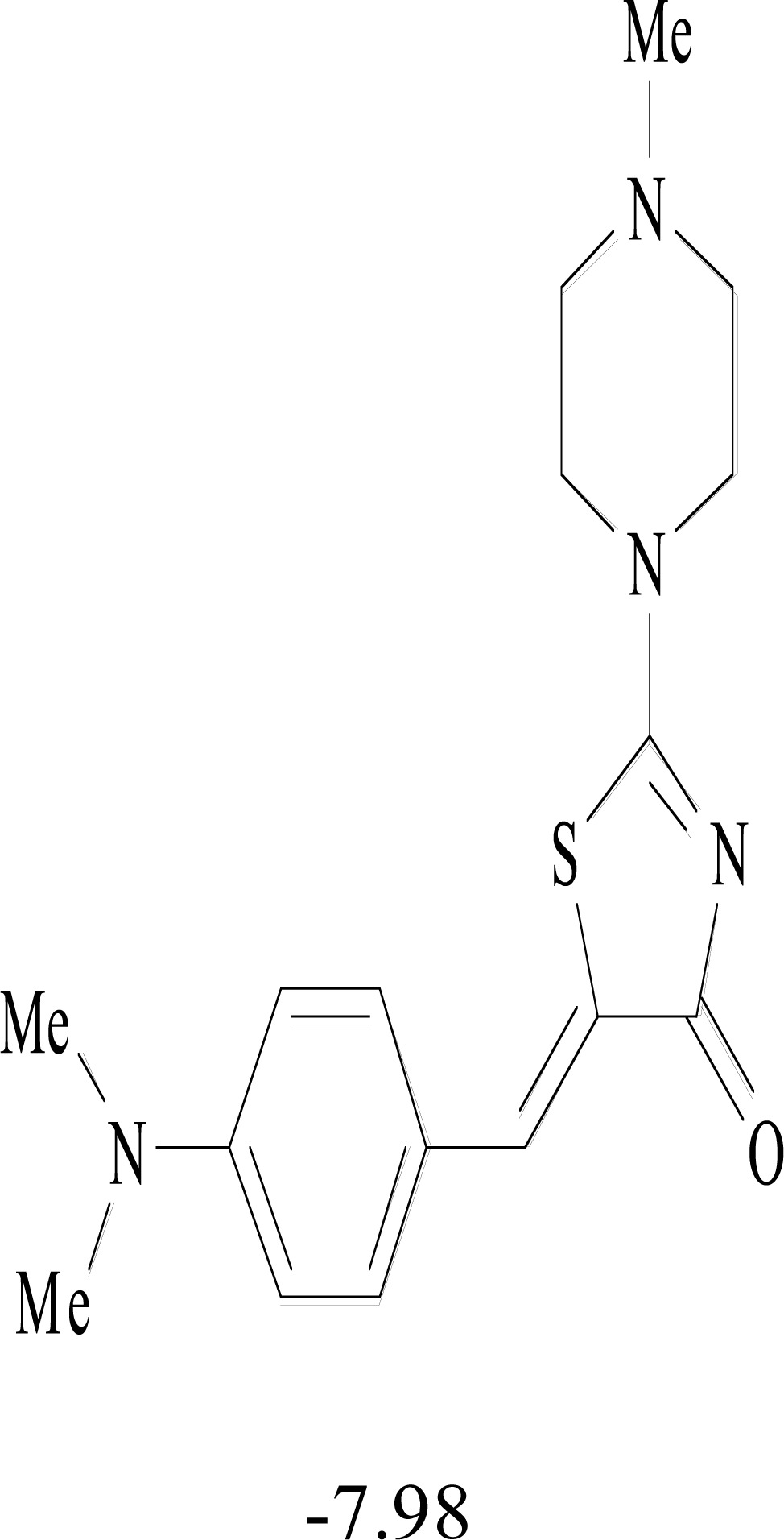
nsP2	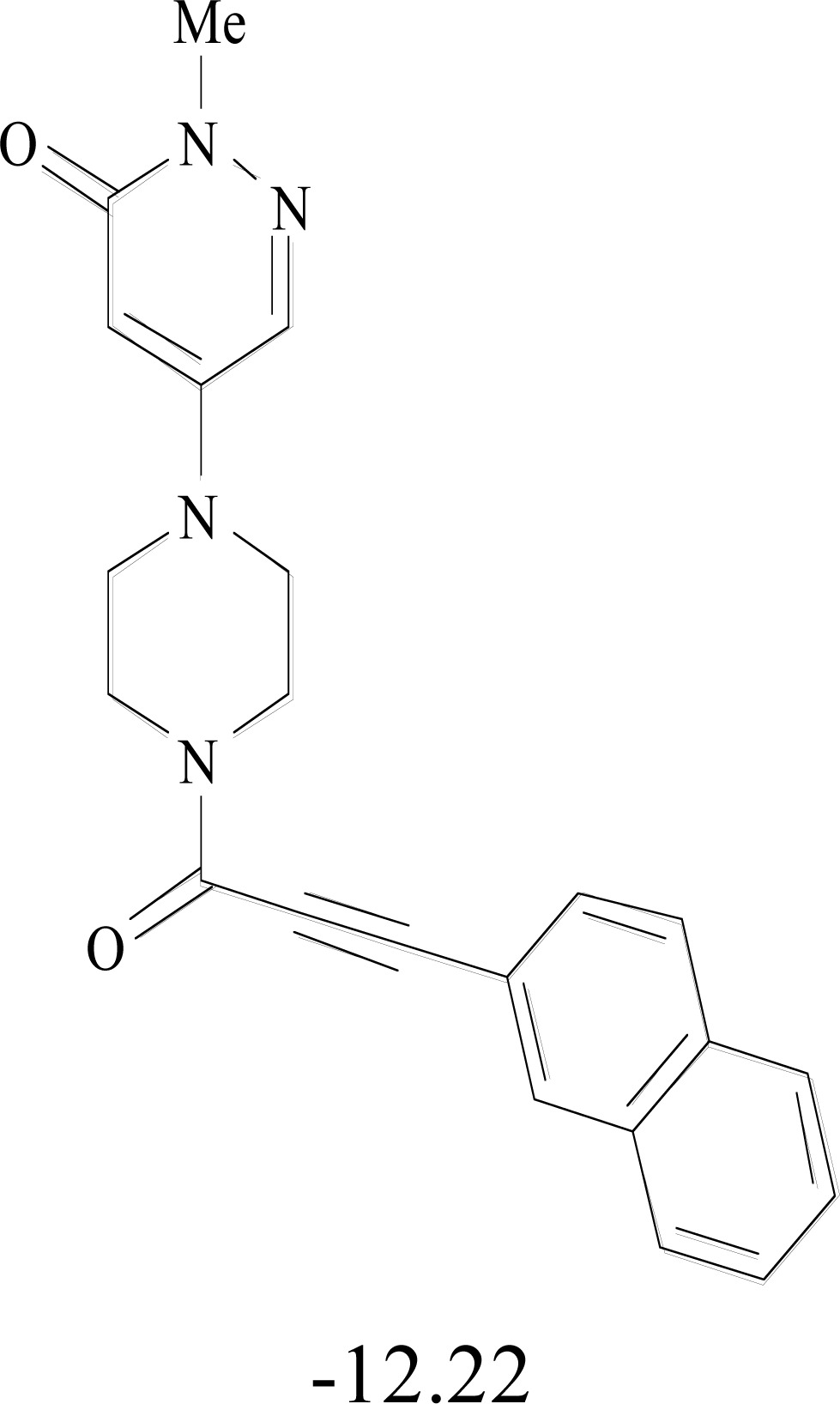	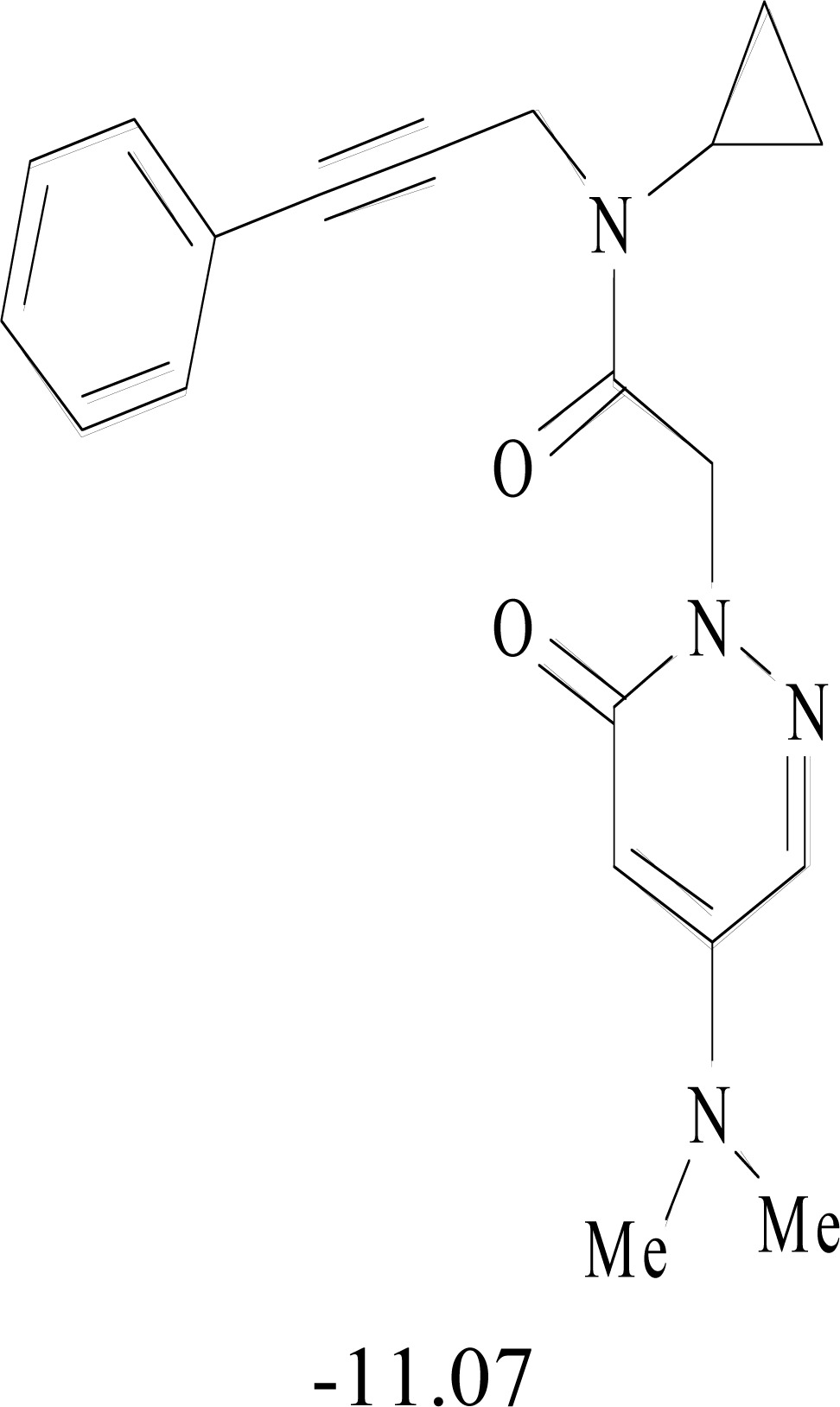	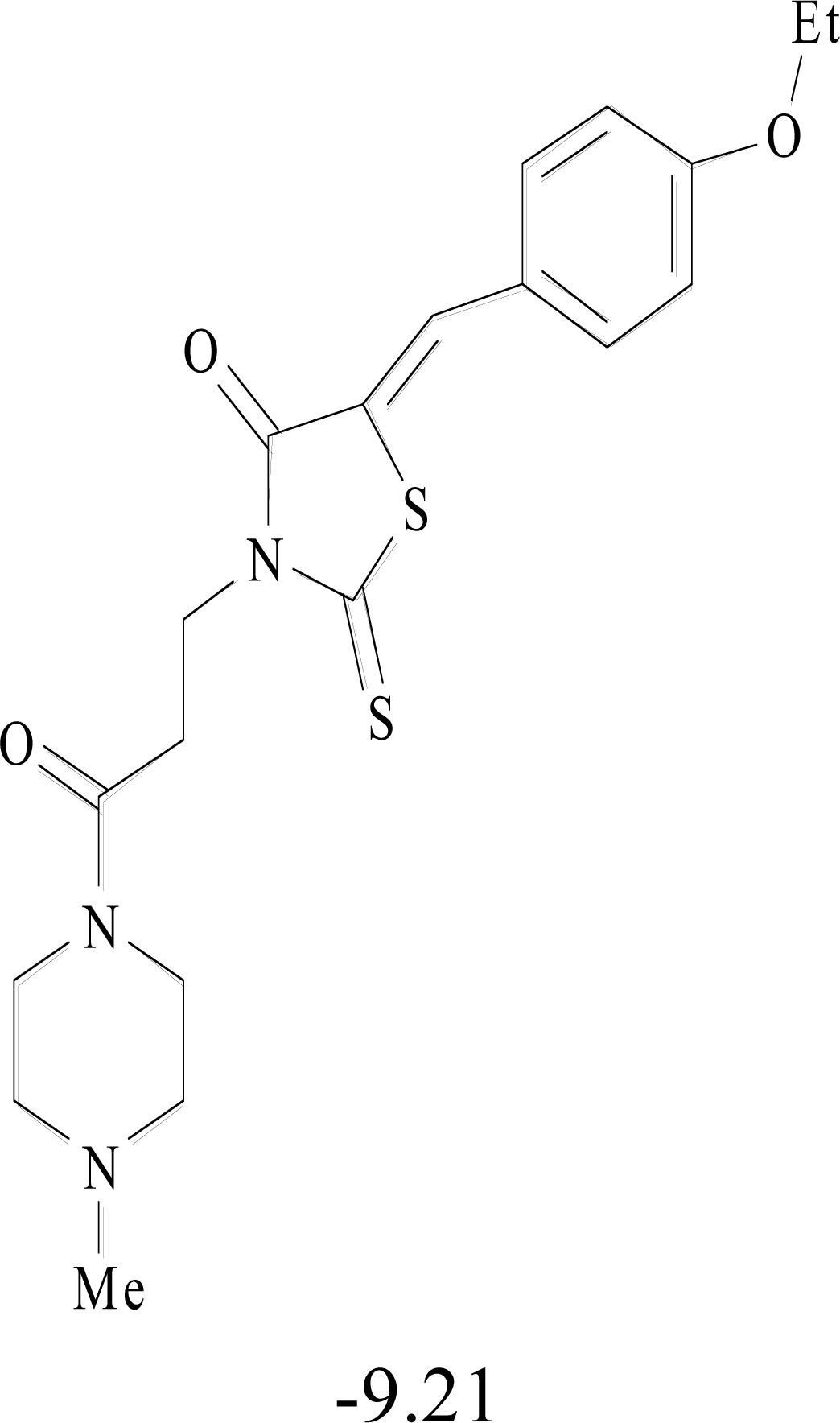	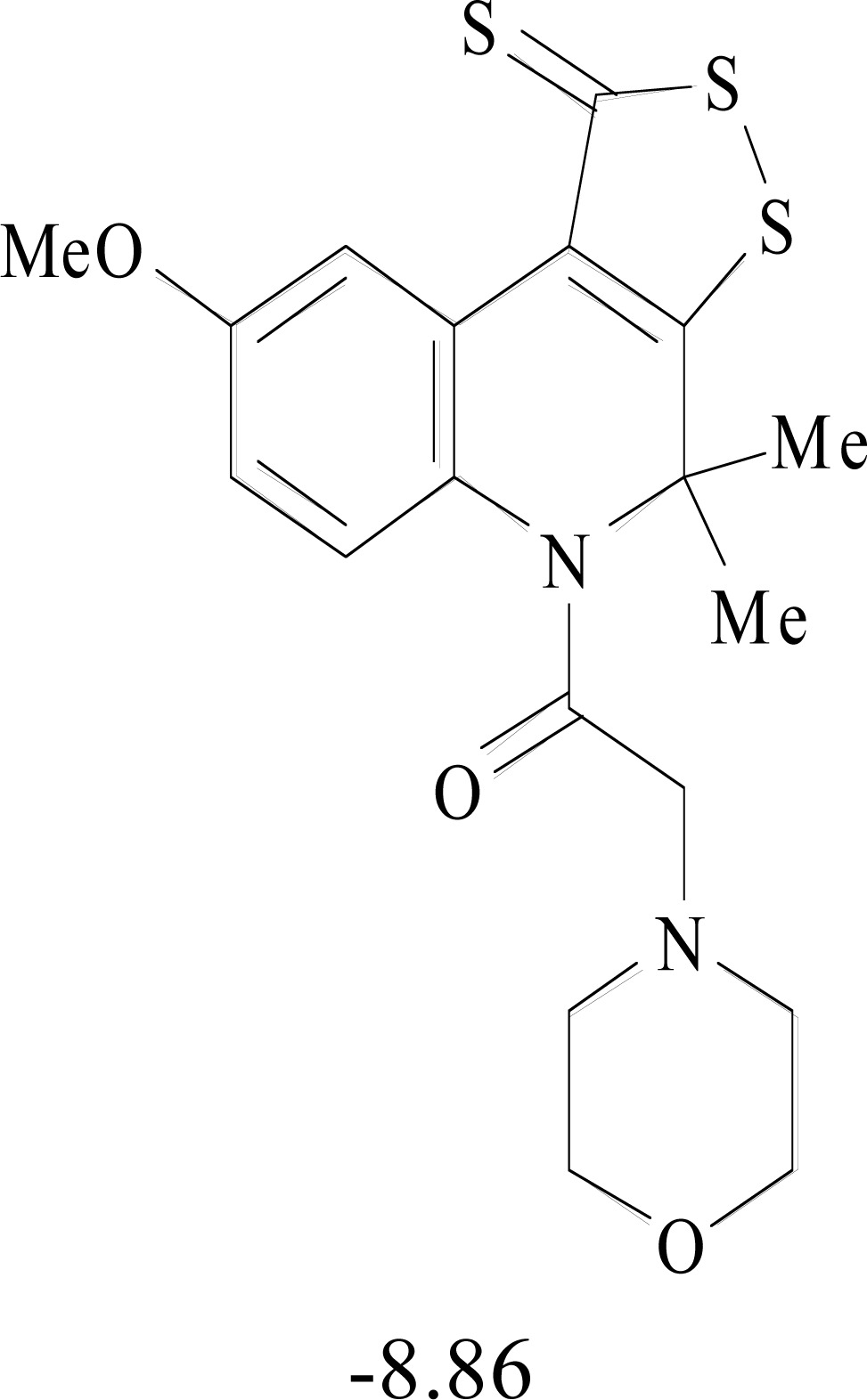	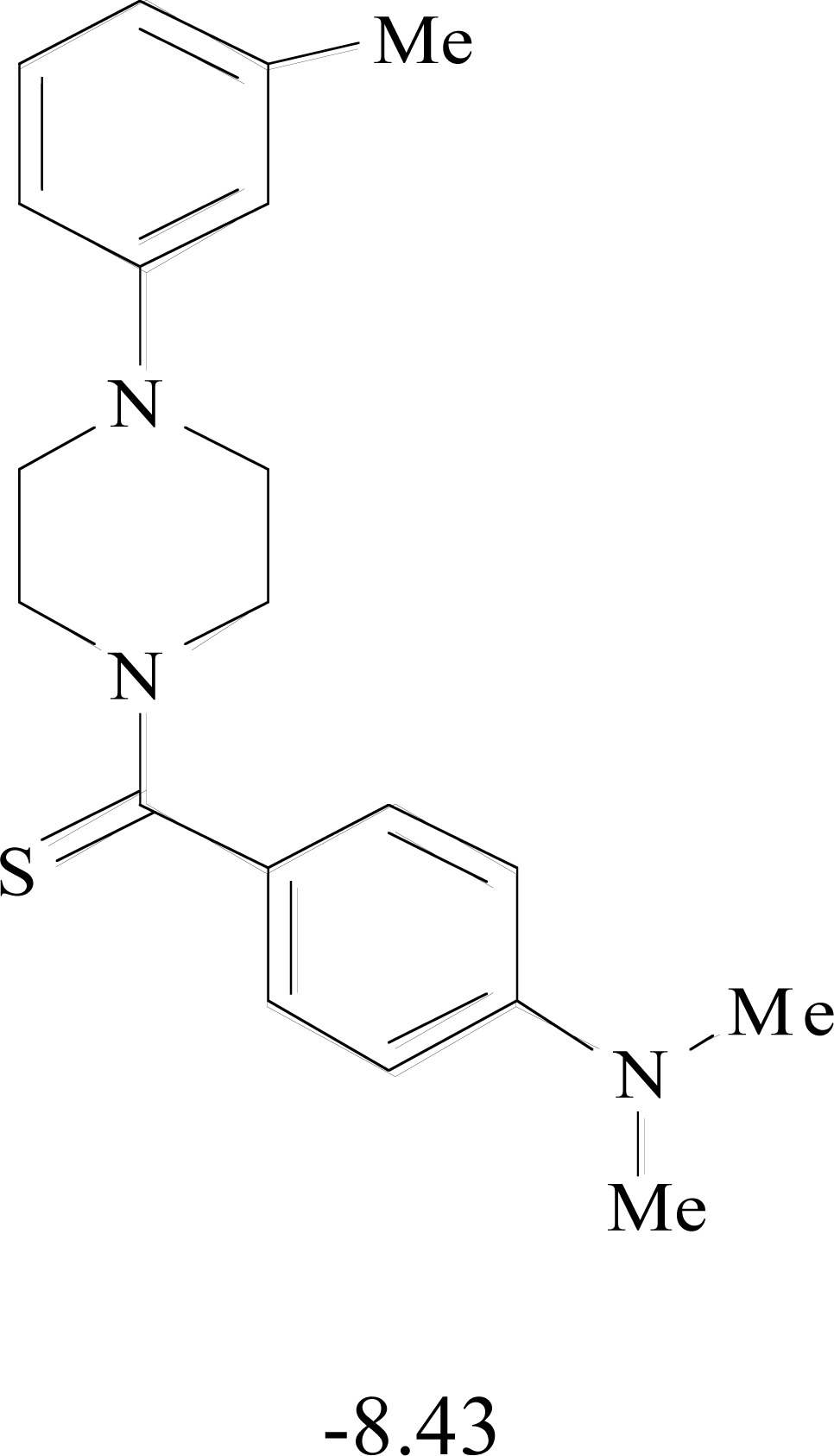
nsP3	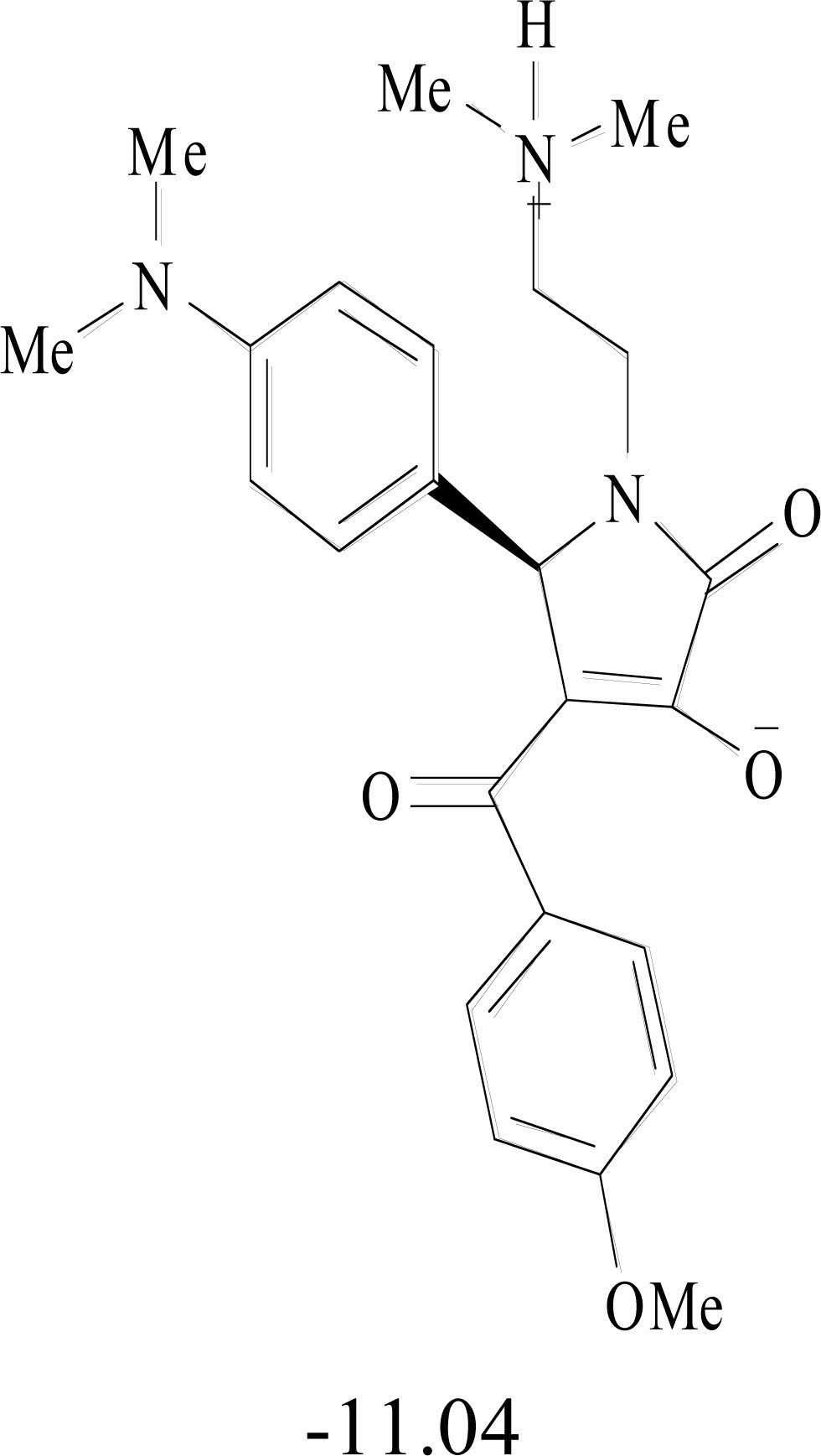	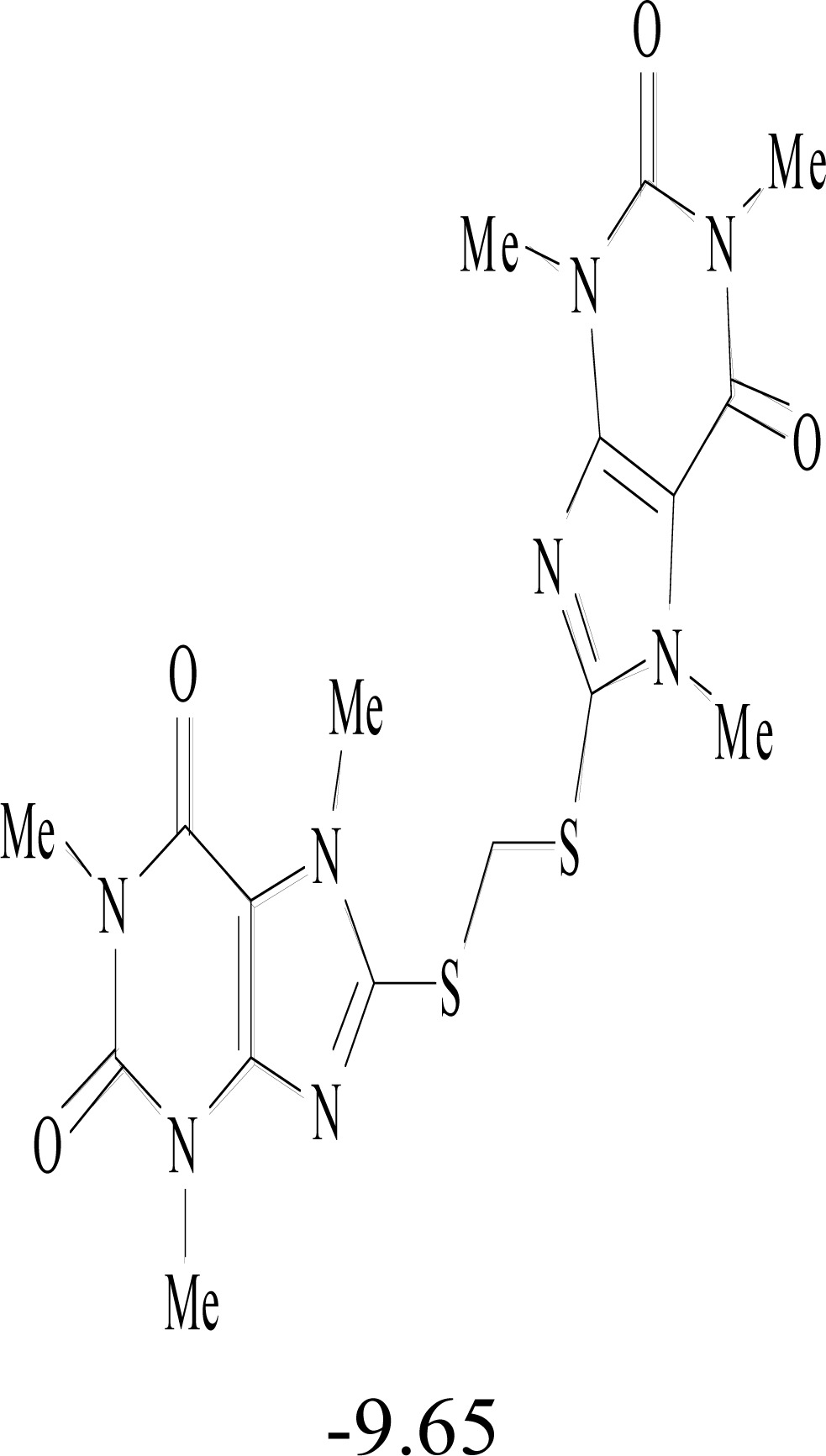	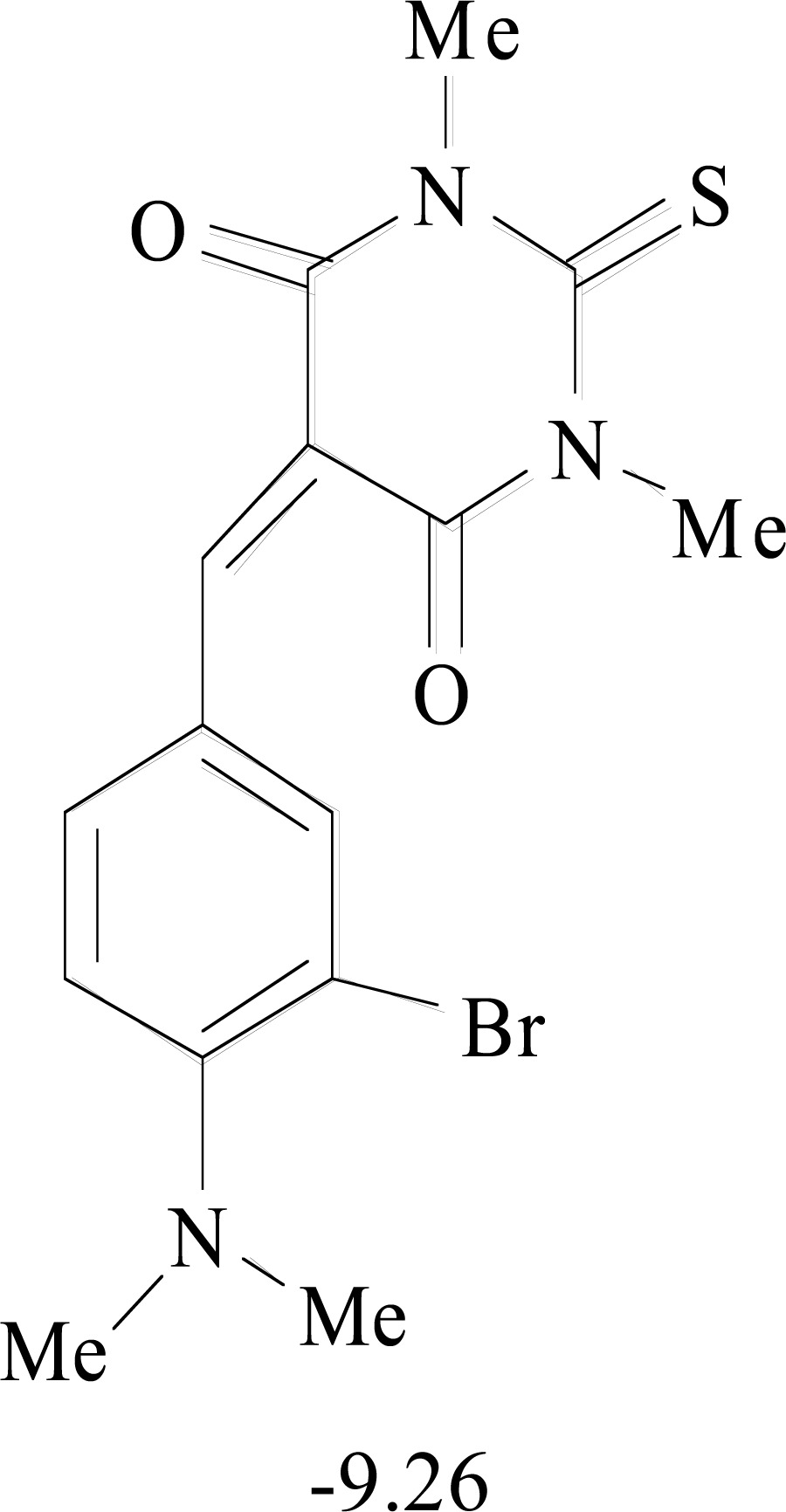	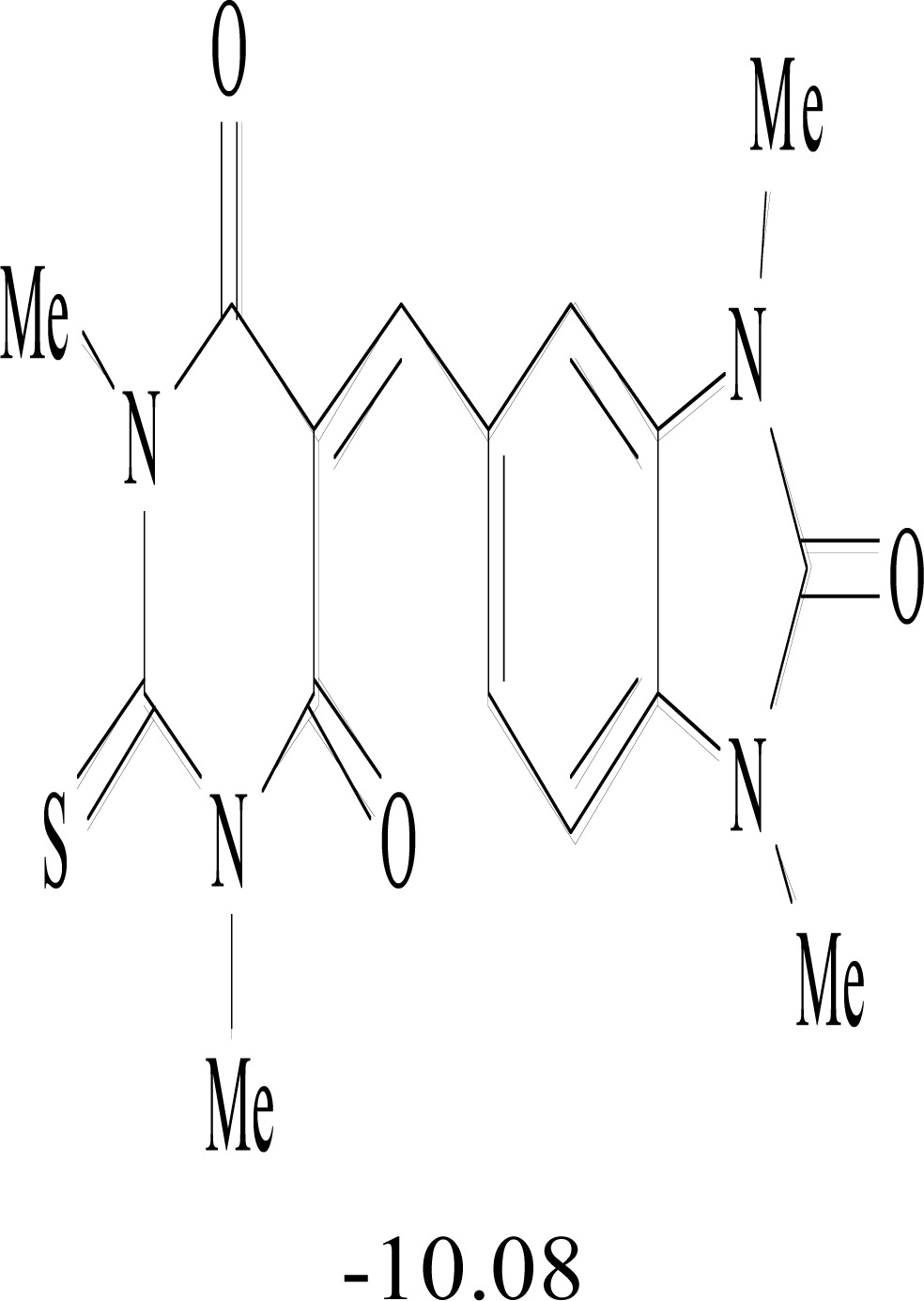	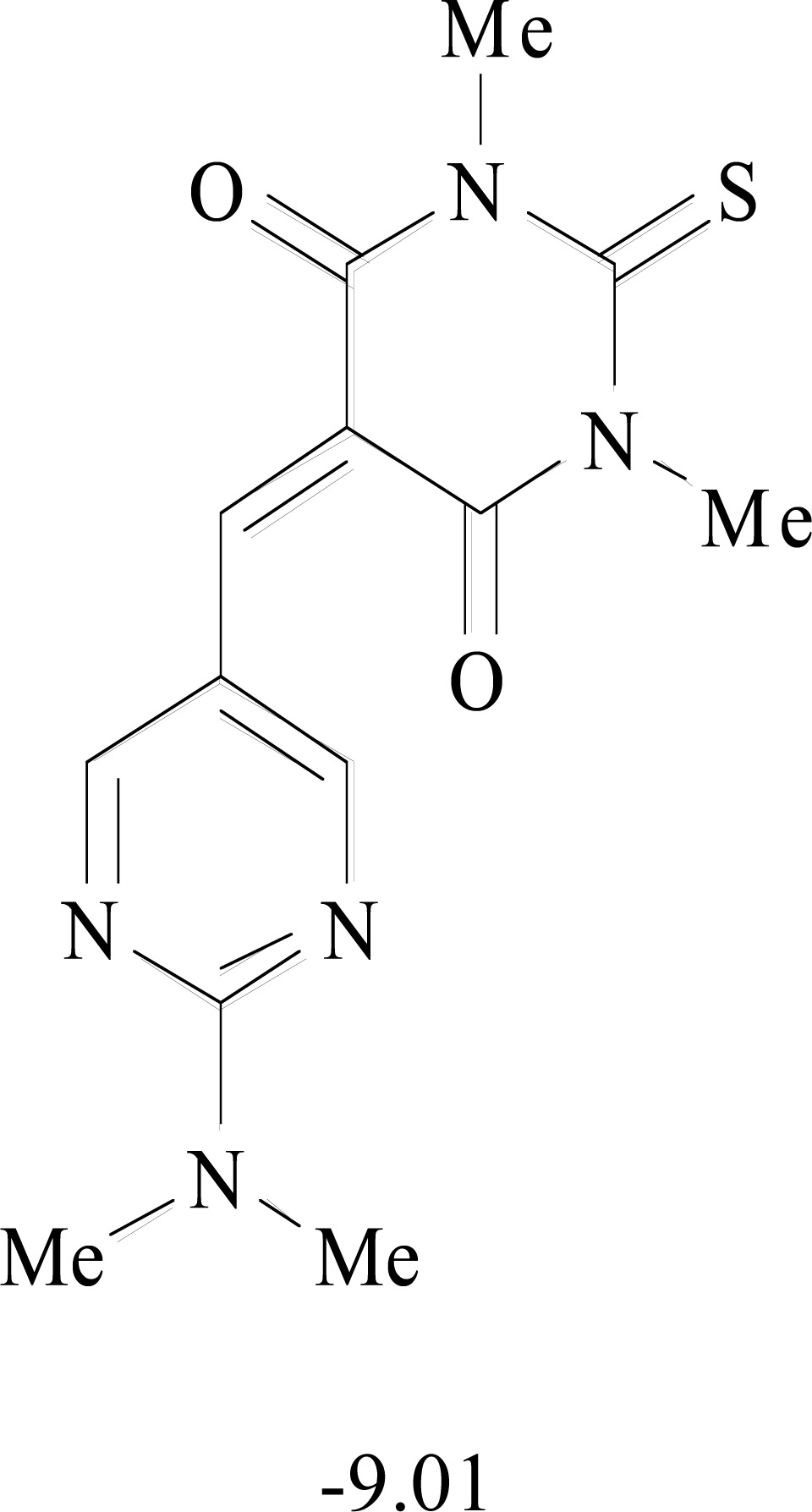
nsP4